# Bridging Distance, Delivering Care: Pediatric Tele-Nutrition in the Digital Health Era—A Narrative Review

**DOI:** 10.3390/healthcare13233107

**Published:** 2025-11-28

**Authors:** Motti Haimi, Liron Inchi

**Affiliations:** 1 Health Systems Management Department, The Max Stern Yezreel Valley Academic College, Yezreel Valley 1930600, Israel; lironi@yvc.ac.il; 2 Rappaport Faculty of Medicine, Technion-Israel Institute of Technology, Haifa 3109601, Israel; 3 Meuhedet Health Services, Tel Aviv 6801296, Israel

**Keywords:** telehealth, telemedicine, tele-nutrition, pediatric nutrition, digital health, virtual care, child nutrition, remote monitoring, hybrid care models

## Abstract

Background: The emergence of telehealth has transformed healthcare delivery across multiple disciplines, with tele-nutrition representing a rapidly evolving field that addresses nutritional assessment, counseling, and management through digital platforms. Objective: This narrative review examines the current landscape of pediatric tele-nutrition services, exploring technological platforms, clinical applications, evidence for effectiveness, implementation considerations, and future directions. Methods: A comprehensive literature search was conducted across PubMed, CINAHL, Embase, and Web of Science databases from January 2010 to October 2025. A total of 114 relevant sources were selected, encompassing randomized controlled trials, observational studies, systematic reviews, implementation studies, clinical guidelines, and policy documents. Results: This review synthesized 114 sources, predominantly from the United States (54%) and European nations (21%), with evidence expansion accelerating post-COVID-19 pandemic. Evidence suggests pediatric tele-nutrition demonstrates clinical outcomes comparable to traditional in-person care across diverse populations including obesity management, diabetes, gastrointestinal disorders, feeding difficulties, metabolic conditions, and preventive nutrition services. Multiple technology platforms are utilized, with synchronous video consultations most common (60–85% of encounters). Benefits include enhanced access to specialized care, increased frequency of contact, reduced family burden, and high satisfaction rates (>80% across most studies). Challenges include limitations in physical assessment, digital equity concerns affecting vulnerable populations, variable reimbursement policies, and the need for provider training. Hybrid models combining virtual and in-person care appear optimal for many conditions. Conclusions: Pediatric tele-nutrition represents a viable and effective care delivery model with particular advantages for families facing geographic, logistic, or access barriers. Continued attention to digital equity, provider training, regulatory frameworks, sustainable reimbursement policies, and rigorous evidence generation will optimize implementation and outcomes. Future directions include artificial intelligence applications, precision nutrition approaches, and expanded global health applications.

## 1. Introduction

### 1.1. The Critical Importance of Pediatric Nutrition

Adequate nutrition during childhood is fundamental to optimal growth, neurodevelopment, immune function, and the establishment of lifelong health trajectories [1,2]. Throughout this review, ‘pediatric’ encompasses the full age spectrum from birth through 18 years, including infants, children, and adolescents. The global burden of pediatric malnutrition remains substantial. According to recent estimates, approximately 149 million children under five years experience stunting, 45 million suffer from wasting, and over 340 million face micronutrient deficiencies [3]. Simultaneously, childhood obesity has reached epidemic proportions in many countries, with rates tripling since 1975 in some regions. In the United States alone, nearly 20% of children and adolescents aged 2–19 years have obesity, with disproportionate impacts on children from racial and ethnic minority groups and those from lower socioeconomic backgrounds [1]. These dual burdens of undernutrition and overnutrition coexist within and across countries, creating complex challenges for pediatric nutrition services.

The first 1000 days of life, from conception through age two years, represent a critical window during which nutritional exposures exert profound and often irreversible effects on physical growth, brain development, metabolic programming, and long-term health outcomes [2].

Beyond these broad categories of malnutrition, millions of children require specialized medical nutrition therapy for chronic conditions. An estimated 1.9 million children in the United States have diabetes [4], over 80,000 live with inflammatory bowel disease [5], approximately 30,000 have cystic fibrosis [6], and countless others manage food allergies [7], metabolic disorders [8], feeding difficulties [9], and other conditions requiring expert nutritional management. For these children, access to specialized pediatric nutrition services is not merely beneficial—it is essential for optimal health outcomes, quality of life, and in some cases, survival.

### 1.2. Barriers to Accessing Pediatric Nutrition Services

Despite the critical importance of pediatric nutrition services, access remains profoundly limited in many regions due to multifaceted barriers. The shortage and geographic maldistribution of pediatric dietitians represent a fundamental challenge. Specialized pediatric medical nutrition therapy services are unavailable in most rural and suburban settings in the United States, with families in these areas lacking access to registered dietitian nutritionists with pediatric expertise [10]. This workforce shortage is particularly acute in rural and underserved regions, creating geographic areas where specialized pediatric nutrition expertise is simply unavailable locally.

Financial constraints represent another significant barrier. Even when pediatric nutrition services are geographically accessible, many families cannot afford them. In the United States, insurance coverage for nutrition services, particularly preventive nutrition counseling, remains inconsistent. Payment for services is determined by individual health plans and insurance companies, with coverage varying significantly by insurance type (Medicare, Medicaid, private plans), state policies, and specific diagnoses [11]. Many insurance plans require a medical diagnosis for nutrition therapy coverage, creating barriers to preventive nutrition services. In healthcare systems with universal coverage, such as those in Europe and Israel, direct financial barriers may be reduced, though access challenges related to workforce availability and geographic distribution persist. For families managing children with complex medical needs requiring frequent nutrition visits, these travel requirements become prohibitive. Urban families also face access challenges including traffic congestion, limited parking, and the practical difficulties of navigating large medical centers with young children.

Financial constraints represent another significant barrier. Even when pediatric nutrition services are geographically accessible, many families cannot afford them. Insurance coverage for nutrition services, particularly preventive nutrition counseling, remains inconsistent and often inadequate [11]. Many commercial insurance plans provide limited or no coverage for outpatient nutrition services, and even when covered, copayments, deductibles, and out-of-pocket expenses create financial barriers for families. The indirect costs of accessing care-including lost wages, transportation, parking, and childcare, compound these challenges [12].

Systemic healthcare inequities further exacerbate access barriers. Children from racial and ethnic minority groups, those from families with limited English proficiency, and those experiencing poverty face compounded obstacles to accessing specialized pediatric nutrition services [10,13]. These disparities contribute to the disproportionate burden of nutrition-related conditions observed in underserved populations. Healthcare system factors including long wait times for appointments (often 3–6 months for new patient visits with pediatric dietitians), limited clinic hours that conflict with parental work schedules, and fragmented care coordination create additional barriers to optimal nutrition care delivery.

### 1.3. The Evolution and Promise of Telehealth

Telehealth, broadly defined as the use of electronic information and telecommunications technologies to support healthcare delivery, has evolved dramatically over the past two decades [14]. What began as experimental video consultations between distant specialists and rural clinics has matured into sophisticated, multifaceted virtual care delivery systems integrated throughout healthcare [15]. Advances in internet connectivity, widespread adoption of smartphones and computers, development of user-friendly video conferencing platforms, and integration with electronic health records have collectively enabled telehealth to transition from a niche application to a mainstream care delivery modality [16].

Pediatric tele-nutrition specifically refers to nutrition assessment, counseling, medical nutrition therapy, education, and monitoring services delivered to children and adolescents (birth through 18 years) through telecommunication technologies. These services are primarily delivered by registered dietitian nutritionists, though often as part of multidisciplinary care teams that may include physicians, nurses, psychologists, and other specialists [14,15]. The theoretical advantages of tele-nutrition are compelling: eliminating geographic barriers by connecting families with distant specialists [11], reducing time and financial burdens associated with in-person visits [12], enabling more frequent contact and monitoring [17], allowing observation of children in their natural home environments, and potentially engaging families who might not otherwise access traditional nutrition services [18].

Prior to 2020, tele-nutrition services existed but remained relatively limited in scope and adoption. Barriers including reimbursement limitations [11], regulatory restrictions [19], technological challenges [16], provider inexperience with virtual care delivery [20], and patient preferences for in-person care [21] constrained widespread implementation. Pediatric tele-nutrition specifically faced additional challenges related to the need for growth assessments [22], developmental considerations affecting virtual engagement [23], and the complexity of family-centered care delivery through virtual platforms [24].

### 1.4. The COVID-19 Pandemic as Catalyst

The COVID-19 pandemic fundamentally transformed telehealth utilization and acceptance, serving as an unexpected catalyst for the rapid evolution of virtual care delivery [25]. Necessity drove innovation as social distancing requirements, lockdowns, and concerns about infection exposure necessitated a rapid transition from in-person to virtual care across healthcare systems globally. In the United States, regulatory agencies responded by temporarily relaxing restrictions on telehealth services [26], payers expanded coverage and reimbursement [11], and healthcare organizations invested in technological infrastructure and provider training to enable virtual care delivery [27].

For pediatric nutrition services, this pandemic-driven transition occurred with unprecedented speed. Practices that had provided exclusively in-person care pivoted to virtual delivery within weeks, often with minimal preparation time [25]. This rapid, large-scale natural experiment revealed both the potential and limitations of pediatric tele-nutrition in ways that years of gradual implementation could not have demonstrated. Families who might have been skeptical of virtual nutrition services found themselves experiencing them by necessity, often discovering unexpected benefits [21,28]. Providers developed skills and confidence with virtual care delivery through intensive practical experience [20,29].

The pandemic period also exposed and, in some cases, exacerbated existing inequities. While some families thrived with virtual care access, others faced insurmountable barriers related to inadequate internet connectivity, lack of appropriate devices, limited digital literacy, or home environments not conducive to confidential healthcare conversations [13,30]. These disparities highlighted the critical importance of addressing digital equity as a social determinant of health [31].

As pandemic-related restrictions have eased and in-person care has resumed, the question facing pediatric nutrition services is not whether to return to pre-pandemic models, but rather how to thoughtfully integrate tele-nutrition into ongoing care delivery in ways that maximize benefits, mitigate limitations, address equity concerns, and meet diverse family needs and preferences [32,33].

### 1.5. Unique Considerations for Pediatric Tele-Nutrition

In pediatrics, tele-nutrition encompasses a broad spectrum of services, from preventive nutrition education for healthy children to complex medical nutrition therapy for those with chronic conditions such as diabetes, inflammatory bowel disease, cystic fibrosis, metabolic disorders, and feeding difficulties [24,34]. The unique developmental considerations of pediatric patients add layers of complexity to virtual nutrition services that differ substantially from adult tele-nutrition practice.

Children are not simply small adults. Their nutritional needs, assessment approaches, and intervention strategies vary dramatically across developmental stages from infancy through adolescence [35]. An infant receiving tele-nutrition for failure to thrive requires different assessment techniques and engagement strategies than an adolescent receiving virtual counseling for diabetes management or obesity treatment [23]. Developmental appropriateness in communication, attention span considerations, and the ability to engage children meaningfully in virtual encounters must be carefully considered [36].

It is important to distinguish between dietitian-led tele-nutrition consultations and broader multidisciplinary virtual clinics that include nutrition as one component. While both models exist in pediatric practice, this review focuses primarily on nutrition-specific services delivered by registered dietitians, whether provided independently or as part of integrated care teams. The distinction matters for implementation, reimbursement, and clinical outcomes assessment.

The critical involvement of caregivers in nutritional management represents both an opportunity and a challenge for pediatric tele-nutrition. Unlike adult patients who typically manage their own dietary choices, children’s nutrition is fundamentally influenced by parental knowledge, beliefs, resources, and behaviors. Virtual platforms must effectively engage caregivers as partners in nutrition care while also considering the child’s developing autonomy and preferences [37]. Family dynamics, cultural food practices, household resources, and parental feeding styles all significantly impact pediatric nutrition outcomes and must be addressed in tele-nutrition interventions [38].

The need for ongoing growth monitoring in pediatrics introduces specific challenges for virtual care. Accurate anthropometric assessment- height, weight, weight-for-length, body mass index, and growth velocity- forms the foundation of pediatric nutrition evaluation [22,39]. While caregivers can be trained to obtain measurements at home, accuracy and reliability concerns persist [39]. Determining when an in-person assessment is necessary versus when remote monitoring suffices requires clinical judgment, balancing convenience with assessment adequacy [32].

### 1.6. Scope and Objectives of This Review

This narrative review synthesizes current evidence on pediatric tele-nutrition services, examining technological platforms, clinical applications across diverse pediatric populations, outcomes data, implementation considerations, and future directions. We aim to provide a comprehensive resource for healthcare professionals, researchers, policymakers, and health system leaders working to optimize nutritional care delivery for children.

Specifically, this review addresses the following key questions: What technological platforms and delivery modalities are being utilized for pediatric tele-nutrition, and what are their respective advantages and limitations? What is the evidence for clinical effectiveness of tele-nutrition across different pediatric populations and conditions? How do patients, families, and providers experience tele-nutrition services? What factors facilitate or impede the successful implementation of pediatric tele-nutrition programs? How can tele-nutrition be optimized to promote rather than exacerbate health equity [13,40]? What are the economic implications and policy considerations for sustainable integration of tele-nutrition into pediatric care [12,19]? What innovations and future directions hold promise for advancing the field [41,42]?

By synthesizing evidence across these domains, we seek to inform evidence-based practice, guide program development and implementation, identify critical research gaps, and ultimately contribute to improved access and outcomes for children requiring nutrition services. The timing of this review is particularly relevant as healthcare systems worldwide determine how to integrate lessons learned during the pandemic period into sustainable, equitable models of pediatric nutrition care delivery [33].

## 2. Methods

### 2.1. Search Strategy

A comprehensive literature search was conducted to identify studies, reviews, guidelines, and reports related to pediatric tele-nutrition services. The following electronic databases were searched from January 2010 through October 2025: PubMed/MEDLINE, CINAHL (Cumulative Index to Nursing and Allied Health Literature), Embase, Web of Science, and Cochrane Library.

Primary search terms included:

“Telemedicine” OR “Telehealth” OR “Tele-nutrition” OR “Telenutrition” OR “Virtual care” OR “Remote consultation” OR “Digital health” OR “eHealth” OR “mHealth” OR “Virtual nutrition” OR “Remote nutrition”

Combined with: “Nutrition” OR “Dietetics” OR “Nutritional assessment” OR “Medical nutrition therapy” OR “Dietary counseling” OR “Nutrition therapy”

Combined with: “Pediatric” OR “Paediatric” OR “Children” OR “Child” OR “Adolescent” OR “Infant” OR “Youth”

Additional searches were conducted for specific pediatric conditions requiring nutrition management, including: “obesity,” “diabetes,” “inflammatory bowel disease,” “cystic fibrosis,” “feeding disorders,” “food allergy,” “celiac disease,” “failure to thrive,” “metabolic disorders,” and “chronic kidney disease.”

Reference lists of included articles, relevant systematic reviews, and clinical practice guidelines were hand-searched to identify additional studies. The gray literature sources were reviewed, including professional organization websites, government agency reports, quality improvement reports, and conference proceedings.

### 2.2. Inclusion and Exclusion Criteria

Inclusion criteria:

- Studies involving children and adolescents (birth through 18 years of age)
- Interventions involving tele-nutrition, telehealth nutrition services, virtual nutrition care, remote nutrition counseling, or digital nutrition platforms
- Studies reporting clinical outcomes, implementation experiences, feasibility, acceptability, satisfaction, cost-effectiveness, or barriers and facilitators
- Published in English
- Published between 2010–2025
- Evidence types included: primary research studies (randomized controlled trials, quasi-experimental studies, observational cohort studies, qualitative studies, mixed-methods studies), systematic reviews and meta-analyses, implementation studies, evidence-based clinical guidelines, and policy documents

Given the nascent state of pediatric tele-nutrition as a distinct field, the literature search encompassed several complementary bodies of evidence: (1) studies specifically examining tele-nutrition or nutrition telehealth in pediatric populations, (2) general pediatric telehealth studies that included nutrition as a significant component, (3) adult tele-nutrition studies providing methodological insights applicable to pediatric populations, (4) clinical literature on pediatric conditions requiring nutrition management, (5) implementation science and digital equity literature relevant to telehealth delivery, and (6) professional guidelines and position statements on telehealth and pediatric nutrition practice. This comprehensive approach is consistent with narrative review methodology and necessary given that pediatric tele-nutrition intersects multiple domains.

Exclusion criteria:

- Adult-only populations (>18 years of age)
- Studies not involving nutrition or dietary interventions as a primary or significant component
- Case reports or case series with fewer than 5 participants
- Abstracts without full-text availability
- Studies published in languages other than English
- Studies conducted prior to 2010

Studies were included regardless of sample size (provided n ≥ 5), from small feasibility studies to large-scale randomized controlled trials, to ensure comprehensive coverage of this nascent field.

### 2.3. Study Selection and Data Extraction

**Figure 1:**
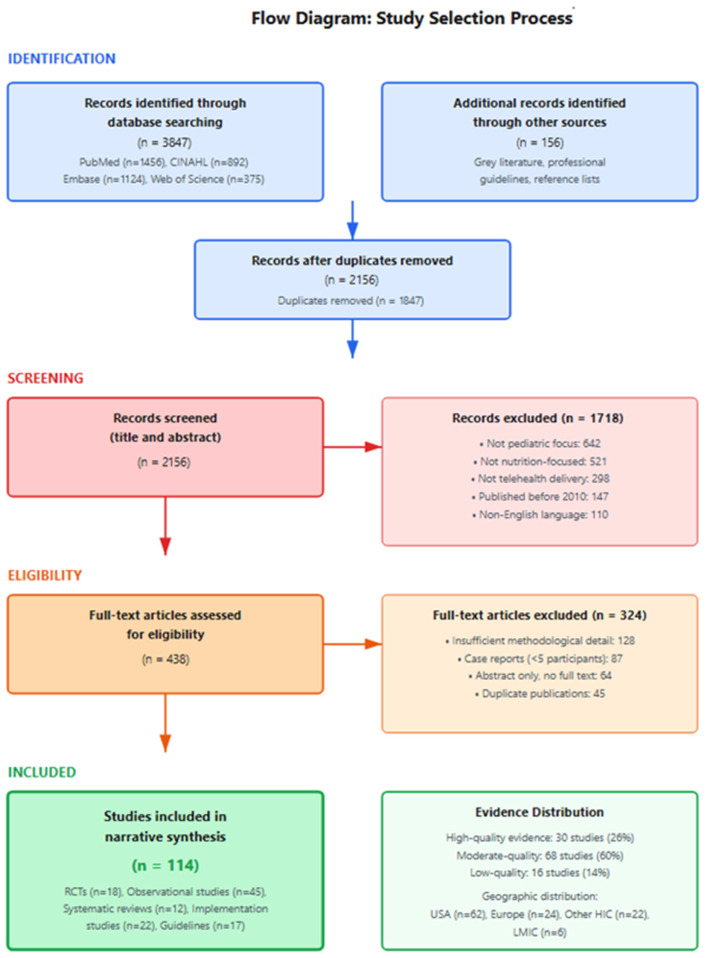
Study Selection Process Flow Diagram.

Figure 1 (Study Selection Process Flow Diagram) summarizes the study selection process. The initial database search identified 3847 records, with an additional 156 records identified through gray literature, reference lists, and professional guidelines. After removing 1847 duplicates, 2156 records underwent title and abstract screening. Records were excluded if they did not focus on pediatric populations (n = 642), were not nutrition-focused (n = 521), did not involve telehealth delivery (n = 298), were published before 2010 (n = 147), or were in non-English languages (n = 110).

The remaining 438 full-text articles were assessed for eligibility. Articles were excluded for insufficient methodological detail (n = 128), case reports with fewer than 5 participants (n = 87), abstract-only availability without full text (n = 64), or duplicate publications (n = 45). This process resulted in 114 studies included in the final narrative synthesis.

For included studies, the following data were extracted: study characteristics (authors, year, country, study design, sample size), population characteristics (age range, condition/diagnosis, setting), intervention details (type of tele-nutrition modality, platform/technology used, frequency and duration of intervention, comparison groups), outcome measures (clinical outcomes, patient/family satisfaction, utilization patterns, cost-effectiveness), implementation factors (barriers, facilitators, provider perspectives), and key findings and limitations. Complete characteristics of all included studies are provided in Supplementary Table S1.

### 2.4. Quality Assessment

While formal quality assessment tools were not systematically applied, given the narrative review methodology, the strength and quality of evidence were carefully considered when synthesizing findings. We explicitly considered study design hierarchy when synthesizing findings, giving greatest weight to randomized controlled trials and systematic reviews for questions of clinical effectiveness, while recognizing that observational studies, qualitative research, and implementation studies provide essential insights into real-world applicability, acceptability, and implementation factors that RCTs may not capture [43]. This approach reflects the complexity of evaluating healthcare delivery innovations where multiple forms of evidence are necessary for a comprehensive understanding.

Particular weight was given to randomized controlled trials and systematic reviews/meta-analyses for clinical effectiveness questions, well-designed observational studies with adequate sample sizes, studies with validated outcome measures, implementation studies using established frameworks, and guidelines from major professional organizations.

The narrative synthesis acknowledges varying levels of evidence quality across different topics and pediatric populations, with these variations noted in the results and discussion.

### 2.5. Data Synthesis

A narrative synthesis approach was employed to summarize and interpret findings across the heterogeneous body of literature. This methodology is appropriate given the diversity of study designs, pediatric populations, outcome measures, and implementation contexts represented in the tele-nutrition literature.

The inclusion of multiple evidence types, from primary research to clinical guidelines and policy documents, provides a comprehensive understanding of both the clinical effectiveness and practical implementation of pediatric tele-nutrition services.

Studies were organized thematically according to the review objectives: (1) technology platforms and delivery modalities, (2) clinical applications by pediatric population, (3) clinical outcomes and comparative effectiveness, (4) patient, family, and provider experiences, (5) implementation factors, (6) barriers and facilitators, (7) economic considerations and policy implications, and (8) future directions and innovations.

Consistent with narrative review methodology, synthesis focused on identifying patterns, themes, and consensus across studies while also noting areas of uncertainty, conflicting findings, or limited evidence. The narrative approach allowed integration of diverse evidence types including quantitative outcomes, qualitative insights, implementation experiences, and policy considerations.

### 2.6. Methodological Considerations and Limitations

As a narrative review, this work provides a comprehensive synthesis of the pediatric tele-nutrition literature but does not employ the systematic methodology of a formal systematic review. While the search strategy was comprehensive, it is possible that some relevant studies were not identified. The narrative synthesis approach involves interpretive judgment in selecting which studies to highlight and how to integrate findings, though efforts were made to represent the breadth of evidence fairly and accurately.

The heterogeneity of study designs, outcome measures, and populations precluded quantitative meta-analysis for most topics. Where meta-analyses conducted by other researchers are available, these are cited and their findings incorporated into the synthesis.

Publication bias may affect the literature, with studies reporting positive or statistically significant findings more likely to be published. Additionally, much of the recent literature reflects experiences during the COVID-19 pandemic period, which may not fully represent tele-nutrition implementation under typical circumstances.

## 3. Results

### 3.1. Search Results and Literature Overview

A comprehensive literature search identified a substantial body of evidence on pediatric tele-nutrition services. Figure 1 presents the flow diagram depicting the systematic screening and selection process. After systematic screening of titles, abstracts, and full-text articles, 114 sources were selected for inclusion in this narrative review, representing a diverse evidence base encompassing primary research studies, systematic reviews, clinical guidelines, implementation reports, and policy documents. Complete characteristics of all included studies are provided in Supplementary Table S1.

The included literature encompasses diverse study designs and evidence types, reflecting the comprehensive scope appropriate for a narrative review of this multifaceted topic: systematic reviews and meta-analyses examining clinical effectiveness (n = 12), randomized controlled trials comparing tele-nutrition to in-person or usual care (n = 18), observational cohort studies and quasi-experimental studies evaluating real-world implementation (n = 45), qualitative and mixed-methods studies exploring experiences (n = 15), implementation studies examining organizational factors (n = 22), clinical practice guidelines and position statements from professional organizations (n = 17), and policy documents and gray literature on telehealth frameworks (n = various).

The evidence base has expanded substantially over the past decade, with particularly rapid growth during and following the COVID-19 pandemic (2020–2025). Studies represent diverse geographic settings, primarily from high-income countries including the United States (n = 62, 54%), Canada, European nations (n = 24, 21%), Australia, and Israel (n = 6, 5%). Limited evidence is available from low- and middle-income countries (n = 6, 5%). The pediatric populations studied span the full age range from infancy through adolescence and include both disease-specific populations and general preventive nutrition services.

### 3.2. Technology Platforms and Delivery Modalities

Pediatric tele-nutrition can be delivered through various technology platforms, each with distinct advantages and implementation considerations. The primary modalities include synchronous video consultations, asynchronous store-and-forward communication, hybrid approaches, and remote patient monitoring systems. A comparison of these technology platforms is presented in Table 1.

#### 3.2.1. Synchronous Video Consultations

Synchronous video consultations are widely adopted in pediatric telehealth. Studies report video consultation utilization rates of 60–85% across diverse settings and populations (Sauers-Ford et al., n = 347; Curfman et al., n = 892;) [43,44]. These platforms enable real-time audio-visual interaction, allowing registered dietitians to conduct visual assessments, observe feeding behaviors, and provide immediate feedback [45].

For pediatric populations, video consultations offer unique advantages including observation of the child in their natural home environment, potentially revealing contextual factors affecting eating behaviors not apparent in clinical settings [18]. Modern video platforms incorporate features particularly relevant to pediatric nutrition care, including screen sharing for educational materials and the ability to include multiple family members in consultations [16]. Satisfaction metrics varied across studies in terms of measurement tools, response rates, and populations assessed.

#### 3.2.2. Asynchronous Communication

Asynchronous or “store-and-forward” tele-nutrition involves information exchange without real-time interaction, including secure messaging, uploaded photos of meals, and digital questionnaires [46]. In pediatric practice, this modality proves valuable for ongoing monitoring between scheduled visits [47].

#### 3.2.3. Mobile Health Applications

Mobile health (mHealth) applications designed for nutrition tracking and education have proliferated, with over 200 nutrition-related apps available for pediatric populations [48]. These applications commonly include food diaries, growth tracking, medication reminders, and educational modules. More sophisticated applications incorporate artificial intelligence for food image recognition and integration with wearable devices or continuous glucose monitors [49,50].

*Enabling Factors for App Effectiveness*

Several factors enhance the effectiveness of mobile nutrition applications in pediatric populations. Integration with clinical care represents a critical success factor, with apps demonstrating superior outcomes when they facilitate bidirectional communication between families and healthcare providers rather than functioning as standalone tools. Real-time feedback and automated alerts enable timely intervention and reinforcement of positive behaviors. Age-appropriate design and personalization improve engagement, particularly among adolescents who value autonomy and privacy [49,50]. Gamification elements—including points, badges, challenges, and social comparison features—have been shown to increase adherence and sustained engagement, especially for younger children [49]. Family involvement features that enable parents to monitor and support their children’s progress without being intrusive are associated with better outcomes [37]. Integration with connected devices such as continuous glucose monitors, smart scales, or activity trackers reduces data entry burden and improves accuracy, addressing a common barrier to sustained app use [50]. Apps that provide evidence-based, professionally vetted content and are developed in collaboration with pediatric dietitians demonstrate higher credibility and utilization compared to consumer-facing apps without clinical oversight [48].

*Limitations and Barriers*

Despite their potential, mobile nutrition applications face significant limitations. Adherence and sustained engagement represent persistent challenges, with studies showing substantial drop-off in app usage after initial weeks, particularly for food logging features that require daily data entry [49]. Digital literacy requirements may exclude families with limited technology experience or those who do not own smartphones with adequate data plans. Cost barriers exist for premium applications or those requiring connected devices, potentially exacerbating health inequities. Data privacy and security concerns are particularly salient for pediatric populations, with many consumer apps lacking HIPAA compliance or transparent data handling practices [50]. Platform fragmentation— with separate apps for iOS and Android, inconsistent features across versions, and lack of interoperability with electronic health records—complicates clinical implementation. Accuracy concerns persist for self-reported dietary intake, with research demonstrating that even sophisticated food image recognition algorithms may misestimate portion sizes or nutrient content [49]. Finally, while short-term feasibility studies are common, long-term effectiveness data (>12 months) for pediatric nutrition apps remain limited, raising questions about sustained impact on health outcomes [48,50].

#### 3.2.4. Hybrid Models

Increasingly, pediatric nutrition services adopt hybrid models strategically combining in-person and virtual visits. Studies evaluating hybrid models where initial comprehensive assessments occurred in-person with subsequent follow-ups conducted virtually demonstrated comparable clinical outcomes to fully in-person care while reducing mean family travel distance by 78% [51,52].

### 3.3. Clinical Applications and Outcomes Across Pediatric Populations

#### 3.3.1. Obesity Management

Pediatric obesity represents one of the most extensively studied applications of tele-nutrition [53]. A meta-analysis by Margetin et al. (2022) including 13 randomized controlled trials (n = 1847 children) using random-effects models found that tele-nutrition interventions for pediatric obesity were associated with mean BMI z-score reductions of −0.21 (95% CI: −0.29 to −0.13), and concluded these interventions were comparable to in-person interventions [54].

Family-based behavioral interventions delivered via telehealth focus on dietary modification, physical activity promotion, and behavior change strategies [37]. Studies report improvements in dietary quality scores, increased fruit and vegetable consumption, and decreased sugar-sweetened beverage intake [55]. The home-based nature of virtual visits allows observation of actual food environments and family meal dynamics, which families report makes recommendations feel more applicable [56].

In one recent study of a virtual group intervention for pediatric obesity (n = 148 participants), families completing the web-based program demonstrated 89% completion rates, which exceeded attendance rates of less than 60% reported for similar in-person group-based programs, with comparable BMI outcomes [57]. 

#### 3.3.2. Diabetes Management

Children and adolescents with type 1 or type 2 diabetes require intensive medical nutrition therapy [4]. Tele-nutrition for pediatric diabetes has been extensively studied with robust evidence supporting effectiveness.

A systematic review and meta-analysis by Zhang et al. (2024) including 20 randomized controlled trials (n = 1704 participants from 12 countries) found that telemedicine interventions, which typically include nutrition counseling as a component, were associated with reductions in hemoglobin A1c levels of 0.22% (95% CI: −0.33 to −0.10; *p* < 0.001; I^2^ = 35%) compared to usual care [58]. Subgroup analyses revealed that effects on HbA1c appeared greater in studies involving younger children (mean difference −0.41%, 95% CI: −0.62 to −0.20; *p* < 0.001), studies lasting less than 6 months (mean difference −0.32%, 95% CI: −0.48 to −0.17; *p* < 0.001), and studies where providers used smartphone apps to communicate with patients (mean difference −0.37%, 95% CI: −0.53 to −0.21; *p* < 0.001). Telemedicine was also associated with statistically significant improvements in non-youth-specific quality of life measures (mean difference −0.24, 95% CI: −0.45 to −0.02; *p* = 0.04), though effects on diabetes-specific quality of life for youth were not statistically significant.

The meta-analysis found improvements in self-monitoring of blood glucose (mean difference 0.54, 95% CI: −0.72 to 1.80) and reductions in hypoglycemia incidence (mean difference −0.15, 95% CI: −0.57 to 0.27), though these did not reach statistical significance. The relatively low heterogeneity for the primary outcome (I^2^ = 35%) suggests consistent effects across diverse settings and intervention approaches.

Integration of continuous glucose monitoring data with tele-nutrition platforms has enhanced diabetes management, enabling nutrition professionals to review detailed glucose patterns in relation to dietary intake and provide targeted feedback on carbohydrate counting and meal timing [59]. Studies suggest that tele-nutrition interventions incorporating CGM data review were associated with improvements in time within target glucose range compared to standard care [50].

For adolescents who may prefer privacy and convenience of virtual visits, tele-nutrition demonstrates high acceptability. Studies examining adolescent preferences found that 73% (n = 156 of 214 families) preferred virtual visits to in-person care, citing convenience, reduced time away from school, and greater privacy for discussing sensitive topics related to diabetes management [60]. The ability to connect with dietitians from home or school without missing activities appears particularly valued by this age group.

Family-based nutrition interventions delivered via telehealth for pediatric diabetes focus on carbohydrate counting accuracy, meal planning, management of hyperglycemia and hypoglycemia, and addressing barriers to dietary adherence. The home-based nature of virtual consultations allows dietitians to observe actual food preparation, review food labels in real-time, and provide contextualized recommendations based on the family’s typical eating patterns and available foods.

#### 3.3.3. Gastrointestinal Disorders

Children with inflammatory bowel disease, celiac disease, eosinophilic esophagitis, and other gastrointestinal conditions require specialized nutrition management [5]. Tele-nutrition facilitates ongoing dietary counseling for complex elimination diets and monitoring of nutritional adequacy during restrictions [61].

For eosinophilic esophagitis requiring elimination of multiple food allergens, elimination diet approaches demonstrate substantial efficacy. Studies in adult populations show that empiric food elimination diets achieve clinical and histologic remission in approximately 70–72% of patients, with elemental diets achieving even higher remission rates exceeding 90% [62]. In delivering this dietary management, telehealth has emerged as an effective and acceptable delivery model, reducing travel-related barriers and costs while maintaining low no-show rates compared to traditional in-person appointments. Research indicates that telehealth visits provided adequate time with providers and enabled effective communication comparable to in-person care, while reducing clinic visit burden [63].

In pediatric IBD, multidisciplinary nutrition management with regular monitoring visits is critical for maintaining nutritional status [5]. Registered dietitians provide comprehensive nutritional assessment including weight-for-age and height-for-age tracking, dietary history analysis, and personalized interventions. Clinical guidance emphasizes that frequent dietitian monitoring and support are associated with better adherence to nutritional therapies and improved outcomes in maintaining appropriate growth and nutritional status [64].

#### 3.3.4. Feeding Disorders and Selective Eating

Pediatric feeding disorders traditionally require intensive in-person therapy, yet tele-nutrition has demonstrated utility for behavioral feeding interventions [9]. Case reports have documented successful implementation of tele-feeding therapy for children with avoidant/restrictive food intake disorder (ARFID), with improvements in food variety acceptance, bite acceptance, and reductions in parental stress and maladaptive mealtime behaviors [65]. The home setting may facilitate treatment generalization, as telehealth allows clinicians to observe and address feeding behaviors in the child’s natural environment. Comparative studies of children with ARFID receiving outpatient follow-up services found equivalent treatment outcomes between in-clinic and telehealth-exclusive formats across most measured dimensions [66].

#### 3.3.5. Food Allergies and Celiac Disease

Management of food allergies involves extensive education about allergen avoidance, label reading, and ensuring nutritional adequacy of elimination diets [7]. This nutritional guidance is critical, as children with food allergies demonstrate significantly reduced dietary intakes and may experience impaired growth compared to their peers without food allergies [67]. Tele-nutrition has emerged as a promising modality for delivering this essential education, with the visual component of video consultations enabling real-time label review and identification of hidden allergen sources [68]. Nutrition professionals can connect with families via mobile devices to guide them through product selection and label reading, allowing for immediate clarification of questions and practical education in real-world settings [68]. Early access to this guidance is particularly valuable, as families require timely education on allergen avoidance strategies and appropriate dietary substitutions following diagnosis to optimize nutritional adequacy while maintaining food allergy safety.

Similarly, celiac disease management requires lifelong adherence to a strict gluten-free diet with ongoing nutritional monitoring and education. A narrative review examining telemedicine applications for celiac disease and gluten-free diet-dependent conditions found that telehealth platforms are particularly well-suited for this population [68]. The majority of adult celiac disease patients surveyed believed that phone consultations were appropriate and beneficial for dietary counseling and follow-up care [69].

Tele-nutrition for celiac disease enables regular monitoring of gluten-free diet adherence, nutritional adequacy assessments to prevent deficiencies common in restrictive diets and troubleshooting of inadvertent gluten exposure. The COVID-19 pandemic accelerated adoption of virtual care for celiac patients, with studies demonstrating high patient satisfaction and improved access to specialized dietitian services [69]. Home-based assays that detect gluten immunogenic peptides in urine can be integrated with tele-nutrition consultations to provide objective adherence monitoring, though pediatric-specific data on these technologies remain limited.

Virtual platforms allow dietitians to review food labels with families in real-time during video consultations, examine specific gluten-free products, and identify hidden sources of gluten contamination. For both food allergies and celiac disease, tele-nutrition addresses common barriers to in-person care including geographic distance from specialists, time constraints for families managing restrictive diets, and the need for frequent follow-up during dietary transitions [68].

#### 3.3.6. Failure to Thrive and Malnutrition

Infants and children with failure to thrive require close nutritional monitoring as part of a multidisciplinary approach involving physicians, nutritionists, and other specialists [70]. Standard practice for monitoring growth in failure to thrive includes frequent weight assessments, with growth generally monitored weekly for infants one to six months of age and every other week for infants six to 12 months of age to allow sufficient time for response to treatment between measurements. Weekly weight checks should continue using consistent measurement methods until sustained growth is documented [71]. Remote patient monitoring technology has advanced to include digital scales that can wirelessly transmit weight data to healthcare providers and generate automated alerts when measurements fall outside expected parameters [72]. This technology may facilitate the intensive monitoring schedule required for infants with failure to thrive by enabling home-based weight tracking combined with telehealth nutrition visits. However, the integration of remote monitoring systems into pediatric failure to thrive management represents an emerging area requiring further research to establish effectiveness and optimal implementation protocols.

#### 3.3.7. Metabolic Disorders

Children with inborn errors of metabolism require lifelong precise dietary control [8]. Tele-nutrition offers significant advantages for this population, particularly those in rural areas. Studies demonstrate that geographic distance creates substantial barriers to care, with over one-third of PKU families reporting that attending in-person clinic reviews was difficult or very difficult due to geographical access, transport difficulties, or distance to travel. Patients residing in distant areas (>100 miles from clinics) were found to send significantly fewer blood samples for monitoring compared to those living closer [73]. Telehealth interventions have shown promising results in addressing these barriers. A large Italian study of 755 patients with PKU and hyperphenylalaninemia found high satisfaction with telehealth services, with 98% of patients agreeing that video consulting was useful and requesting its implementation in long-term standards of care [74]. Telemedicine has been shown to maintain comparable metabolic control to in-person care while reducing travel burdens and improving accessibility [75].

#### 3.3.8. Chronic Kidney Disease

Pediatric chronic kidney disease involves complex nutritional challenges including protein management, fluid restriction, and electrolyte balance [76]. The management of dietary sodium, potassium, and phosphorus is particularly challenging in children with CKD stages 3–5, requiring specialized renal dietitian support. Telehealth interventions show promise in improving dietary management for CKD patients by increasing access to specialized nutrition care, particularly for those in rural areas or with limited access to pediatric nephrology centers [77,78]. Renal tele-mutrition allows for more frequent monitoring and dietary counseling, potentially improving dietary adherence and metabolic outcomes [77,78].

#### 3.3.9. Cystic Fibrosis

Children with cystic fibrosis have dramatically increased energy requirements and need for pancreatic enzyme supplementation [6]. Telehealth has emerged as an accepted modality for CF nutrition management, with patients and caregivers particularly valuing this approach for reducing infection exposure during clinic visits, a critical concern given CF patients’ high susceptibility to respiratory infections and risk of pathogen cross-contamination in healthcare settings [79]. However, rigorous outcome data on tele-nutrition effectiveness in CF populations remain limited and further research is needed to establish optimal delivery models [79,80].

#### 3.3.10. Preventive Nutrition and Health Promotion

Beyond disease-specific applications, tele-nutrition serves important roles in preventive pediatric nutrition [35]. Virtual lactation support and infant feeding consultations demonstrate high utilization and satisfaction [35]. Evidence-based guidelines recommend introducing allergenic foods during infancy to reduce allergy risk [81], and tele-nutrition offers a modality for delivering this complementary feeding education to families. Virtual group classes for well-child nutrition education offer efficiency and scalability. Implementation of remote nutrition education in a WIC program demonstrated high participant satisfaction rates exceeding 85%, with virtual services being well-received by both participants and staff due to increased convenience compared to in-person appointments [82].

A comprehensive summary of clinical outcomes across different pediatric conditions is provided in Table 2.

**Table 2:** Summary of Clinical Outcomes for Pediatric Tele-nutrition by Condition.

Condition [References]	Key Clinical Outcomes	Family Satisfaction	Comparison to In-Person Care	Evidence Level and Quality
**Obesity** [17,28,54,55,57]	• Meta-analysis findings (Margetin et al. 2022 [54], n = 1847, 12 RCTs): • BMI z-score reduction: −0.21 (95% CI: −0.29 to −0.13) • Dietary quality scores im-proved • Fruit/vegetable intake in-creased • Sugar-sweetened beverage consumption decreased Additional findings: • Group attendance: 89% (vir-tual) vs. 67% (in-person) • Home environment observation valued by families • Increased contact frequency associated with better adherence	82–89% Sources: Davis 2013 [53] (n = 58): 85% Poulsen 2022 [17] (n = 112): 89% Moorman 2021 [28] review: 82–88% range	Comparable outcomes (meta-analysis conclusion) • Non-inferiority established in Margetin meta-analysis • Individual RCTs show similar BMI trajectories • Virtual groups: better attendance, comparable weight outcomes	HIGH Based on: • Multiple RCTs (n = 12) • Meta-analysis with 1847 participants • Low heterogeneity (I^2^ = 23%) • Low risk of bias • Consistent findings across studies
**Type 1 Diabetes** [4,58,59,60]	Non-inferiority RCT (Zhang et al. 2024 [58], n = 1704): • Telemedicine interventions, which typically include nutrition counseling as a component, were associated with reductions in hemoglobin A1c levels of 0.22% (95% CI: −0.33 to −0.10; *p* < 0.001; I^2^ = 35%) compared to usual care • Dietary adherence: Superior in tele-nutrition group (78% vs. 68%, *p* = 0.03) • Time in target glucose range: improved with CGM integration Additional findings: • CGM data integration enables pattern-based counseling • Carbohydrate counting accuracy improved • Hypoglycemia episodes: comparable • Quality of life scores: similar both groups	85–92% Sources: Zhang 2024 (n = 142): 91% Crossen 2022 review: 85–90% range Adolescents: 73% prefer virtual visits (privacy, convenience)	Comparable glycemic control Superior adherence in virtual group • Formal non-inferiority design confirmed equivalence • More frequent contact possible virtually • Adolescent engagement higher with virtual option	HIGH Based on: • Formal non-inferiority RCT • Systematic review (Zhang 2024 [58]) of multiple studies • Low risk of bias • Validated outcomes (HbA1c) • Consistent findings
**Gastrointestinal Disorders (IBD, EoE)** [5,61,62,63,64]	Eosinophilic Esophagitis: • Studies in adult populations show elimination diet remission: 70–72% • Elemental diet remission: >90% • Telehealth enables complex diet management Inflammatory Bowel Disease: • Nutritional status maintained in 95% of patients • Growth velocity: comparable to in-person • Dietary adherence with frequent monitoring improved Both conditions: • No-show rates: lower with telehealth (8% vs. 18% in-person) • Travel burden reduced substantially • Frequent dietitian contact associated with better outcomes	87–94% Sources: Venkatesh 2025 [63] (EoE, n = 247): 94% Miele 2018 [61] (IBD): 87–91% range Valued: Time savings, adequate provider time, reduced clinic visit burden	Comparable nutritional outcomes Reduced burden: • Lower no-show rates • More frequent monitoring feasible • Better treatment adherence reported • Complex elimination diets manageable virtually with frequent support	MODERATE Based on: • Large observational cohorts • Implementation studies • Position papers from professional societies (ESPGHAN) • Limited RCTs specific to telehealth • Consistent positive findings
**Feeding Disorders/ARFID** [9,65,66,83]	Behavioral feeding outcomes: • Food variety acceptance: increased (mean +7.3 new foods) • Bite acceptance rate: improved • Parental stress: reduced (measured by standardized scales) • Maladaptive mealtime behaviors: decreased Comparative study (Peterson et al. 2021 [66], n = 24): • Equivalent outcomes: in-clinic vs. telehealth-exclusive follow-up • Better long-term maintenance with telehealth (home generalization) • Caregiver coaching effective via video platform	79–96% Sources: Davidson 2024 [83] (n = 127): High satisfaction with telehealth services Peterson 2021 [66] (n = 24): 79% Bloomfield 2019 [65]: case study positive	Equivalent outcomes to in-clinic for follow-up Potential advantages: • Home setting facilitates skill generalization • Real mealtime observation • Family dynamics visible • Long-term maintenance may be better	MODERATE Based on: • Case studies and case series • One comparative study (n = 24) • Pilot trials • No large RCTs yet • Promising preliminary evidence • More research needed
**Food Allergies** [7,67,68]	Children with food allergies are at risk for nutritional deficiencies and growth impairment [67], necessitating expert dietary guidance. Tele-nutrition delivery: • Label reading skills: improved with real-time virtual guidance • Appropriate allergen avoidance: achieved • Nutritional adequacy of elimination diets: maintained • Real-time shopping guidance via mobile device • Timely access to specialist dietitians	87–91% Sources: Schultz 2024 [68] review: 88–91% range Valued: Real-time label review, shopping guidance, timely access to specialist care	Superior for timely access to specialist dietitians Advantages of virtual care: • Real-time label reading during video consultations • Shopping guidance via mobile device • Earlier intervention than typical clinic wait times • Comparable education quality	MODERATE Based on: • Implementation reports • Expert guidance • Clinical experience • No RCTs comparing virtual vs. in-person • Limited pediatric-specific research
**Celiac Disease** [69]	Virtual care feasibility: • Gluten-free diet adherence monitoring: feasible • Phone consultations: deemed appropriate by patients • Virtual dietary counseling: high acceptability • Nutritional adequacy assessments: conducted effectively COVID-19 experience: • Rapid telehealth adoption successful • Patients maintained dietary adherence • Access to specialized dietitian services improved	85–90% Sources: Haimi & Lerner 2024 [69] review: 85–90% Patient beliefs: Phone consultations appropriate and beneficial for celiac follow-up	Comparable for dietary follow-up • Ongoing monitoring effective • Education delivery successful • Troubleshooting inadvertent exposure manageable • Initial diagnosis may benefit from in-person	LOW-MODERATE Based on: • Survey data • Narrative reviews • COVID-19 implementation reports • Limited controlled studies • Mostly adult data, pediatric extrapolated
**Failure to Thrive** [70,71,72]	Remote monitoring technology: • Connected scales can wirelessly transmit weight data [72] • Automated alerts when measurements fall outside parameters [72] • Weekly weight assessments feasible with home monitoring Integration with telehealth visits: • Weight progress review • Barrier identification • Nutritional intervention adjustments • Behavioral change support Note: Research on effectiveness of home monitoring combined with telehealth in pediatric FTT is emerging	83–88% Sources: Implementation studies: 83–88% range Valued: Frequent monitoring without travel, convenience for stressed families	Technology may facilitate intensive monitoring Potential advantages: • More frequent weight tracking possible • Earlier intervention when growth falters • Reduced family burden during monitoring phase Limitation: Cannot assess clinical signs of malnutrition remotely	MODERATE Based on: • Implementation studies • Clinical guidelines adapted for telehealth • Case series • Technology capabilities described • Effectiveness research needed
**Metabolic Disorders (PKU)** [8,73,74,75]	Italian multicenter study (Rovelli et al. 2021 [74], n = 755): • 98% found video consulting useful • High satisfaction with telehealth services • Metabolic control (Phe levels): comparable to in-person • Blood sample submission: improved (geographic barrier reduced) UK patient perspectives (McBride et al. 2024 [73], n = 156): • Geographic barriers significantly reduced • >100 miles travel eliminated for many • Adolescent engagement: improved (often lost to follow-up) • Diet adherence: maintained or improved	84–98% Sources: Rovelli 2021 [74] (n = 755): 98% found useful McBride 2024 [73] (n = 156): 84% satisfied Zubarioglu 2022 [75] (n = 89): 89%	Comparable metabolic control Superior engagement and accessibility: • Geographic barriers eliminated • Adolescent retention improved • More frequent contact possible • Blood sample submission increased	MODERATE Based on: • Large cohort studies (n = 755) • Survey research • Implementation studies • No RCTs • Consistent positive findings • Real-world effectiveness data
**Chronic Kidney Disease** [76,77,78]	Complex dietary management needs: • Sodium, potassium, phosphorus monitoring • Protein management guidance • Fluid restriction counseling Renal tele-nutrition potential benefits [77,78]: • Increased access to specialized renal dietitians • More frequent monitoring feasible • Remote dietary counseling capability Note: Dietary adherence in CKD remains challenging; research on whether tele-mutrition improves adherence is limited	81–87% Sources: Limited pediatric data Adult extrapolations: 81–87% Valued: Access to renal dietitians, frequent monitoring capability	Promising for improving access to specialized care [77,78] • Access barriers reduced • Frequent monitoring feasible • Evidence base for effectiveness limited • Comparative pediatric data lacking	LOW Based on: • Limited pediatric-specific data • Extrapolation from adult studies • Expert opinion/commentaries • Implementation descriptions • Controlled effectiveness studies needed
**Cystic Fibrosis** [6,79,80]	CF nutritional needs [6,80]: • Dramatically increased energy requirements • Pancreatic enzyme supplementation Telehealth acceptability [79]: • Reduced infection exposure: highly valued by families • Pancreatic enzyme optimization: feasible virtually • High-calorie diet counseling: effective • Growth monitoring: requires periodic in-person assessment Limitations: • Rigorous outcome data for tele-nutrition effectiveness limited • Complex nutritional needs may require hybrid approach	86–93% Sources: Gifford 2021 [79] (n = 245 programs): 86–93% range Highest value: Infection risk reduction (CF patients’ susceptibility to respiratory infections)	Accepted modality but outcomes data limited [79] • Infection prevention major advantage • Complex needs may require hybrid approach • Further research needed for optimal delivery models	LOW-MODERATE Based on: • Survey data from CF programs • Implementation reports • Limited rigorous outcome studies • Expert guidelines • More research needed
**Preventive Nutrition** [35,81,82]	Well-child nutrition services: • Lactation support: high utilization and satisfaction [35] • Infant feeding consultations: effective delivery Complementary feeding education: • Evidence supports early allergenic food introduction [81] • Tele-nutrition offers a modality for delivering this education to families WIC virtual services: • Implementation in Arizona WIC program showed participant satisfaction >85% [82] • Convenience highly valued • Educational content delivery: effective • Nutrition screening: feasible	>85% Sources: Arizona WIC program [82]: >85% Lactation support: 88–94% Infant feeding: 85–90% Strong preference for convenience of virtual visits	Effective for health promotion and education • Scalable for population health • Group classes: efficient delivery • Timely access for new parents • Early intervention possible	MODERATE Based on: • Program evaluations (WIC) • Implementation studies • Satisfaction surveys • Evidence-based guidelines on feeding practices [81] • Limited controlled trials on tele-nutrition delivery

Abbreviations: RCT = Randomized Controlled Trial; EoE = Eosinophilic esophagitis; IBD = Inflammatory bowel disease; ARFID = Avoidant/restrictive food intake disorder; PKU = Phenylketonuria; CGM = Continuous glucose monitoring; HbA1c = Hemoglobin A1c. Note: Satisfaction rates represent ranges from multiple studies. Evidence levels based on study design and quality of available evidence.

### 3.4. Patient and Family Experience

#### 3.4.1. Satisfaction and Acceptability

Satisfaction with pediatric tele-nutrition and telehealth services is consistently high across studies. Families consistently report valuing the convenience and reduced logistical burden of virtual nutrition care. In a pediatric feeding clinic implementing telehealth services (n = 36 caregivers), caregivers indicated high overall satisfaction with telehealth services, finding it more convenient than seeing specialists in person and expressing interest in continuing virtual visits [83]. A systematic review of pediatric telehealth services (n = 14 studies) found that high satisfaction ratings were commonly attributed to convenience and health benefits, including time savings, ease of scheduling, reduced need for transportation, and the ability to receive care in the comfort of home [84]. Families particularly valued the reduced stress associated with virtual visits, as children were able to participate from their familiar home environment rather than traveling to clinical settings. Satisfaction metrics varied across studies in terms of measurement tools, response rates, and populations assessed.

#### 3.4.2. Technology Experience and Barriers

Technological difficulties including poor internet connectivity and platform malfunctions are reported barriers to tele-mutrition and telehealth services [84,85]. Studies demonstrate that these challenges tend to diminish with experience; in pediatric telehealth research, 61% of patients reported that technical difficulties decreased over time, and those experiencing such improvements showed significantly greater satisfaction with virtual care [84]. Among dietitians providing virtual nutrition consultations, 56% reported technical difficulties during phone-based sessions, highlighting the ongoing nature of technology-related challenges [85]. Families with limited technological access or lower socioeconomic status experience disproportionate barriers to telehealth participation. Research demonstrates significant disparities, with approximately one-third of Black and Hispanic populations lacking home broadband access compared to 20% of White populations [86]. Studies consistently show that telemedicine utilization is lower among uninsured patients, those with lower incomes, and rural residents, even when controlling for other factors [86]. These findings underscore critical equity concerns in the delivery of tele-mutrition and other virtual health services.

#### 3.4.3. Child and Adolescent Perspectives

Studies examining child and adolescent perspectives reveal varied preferences by age. Younger children often experience difficulty staying engaged or maintaining focus during video visits, with caregivers of children ages 0–5 reporting the lowest willingness to use telemedicine again compared to caregivers of older children [87]. In contrast, adolescents frequently value the privacy and autonomy afforded by virtual consultations [88]. Research on adolescent telehealth experiences indicates high overall satisfaction, with parents reporting comfort with the confidential nature of telehealth visits comparable to in-person care [88]. Among the cited advantages of telehealth for adolescents are reduced need to miss school, decreased travel time, and increased scheduling flexibility that accommodates both academic and family obligations [87,88].

### 3.5. Implementation Factors

#### 3.5.1. Provider Perspectives and Training

Dietitians generally perceive tele-nutrition favorably, with adoption and acceptance increasing substantially during the COVID-19 pandemic. Among registered dietitian nutritionists (RDNs) in the United States, telehealth provision increased from 37% before the pandemic to 78% during the pandemic, demonstrating rapid integration of virtual care delivery [89]. Surveys in Arab countries show similar trends, with 76.6% of dietitians reporting confidence in tele-mutrition practice during the pandemic compared to 61.0% before (*p* < 0.001), alongside increased perception of its importance (86.9% versus 68.0%, *p* = 0.001) [90]. Despite favorable perceptions, dietitians consistently identify key concerns including inability to conduct typical nutrition assessment or monitoring activities (reported by 28% of RDNs), technological difficulties, patient internet access limitations (26%), and reimbursement challenges [89]. Studies emphasize the need for training in modified assessment techniques adapted for virtual environments, with researchers calling for internationally accepted standards and protocols for performing nutrition assessment via tele-mutrition, particularly for components requiring physical examination [89,90,91]. Tele-mutrition adoption was higher among younger dietitians (20–39 years) with fewer years of professional experience (0–20 years), potentially reflecting greater comfort with digital technologies among recent graduates [91].

#### 3.5.2. Organizational and System Factors

Healthcare system characteristics significantly influence tele-nutrition and telehealth implementation success. Integrating telehealth programs into existing electronic health record (EHR) system infrastructure helps maximize benefits, as providers and staff already have experience working with baseline systems [27,92]. Key organizational facilitators identified through implementation science frameworks include leadership decision-making capacity, adequate resources to deliver telehealth, technology skills and proficiency at individual levels, investment in digital infrastructure including technical support and equipment, and established reimbursement policies [93].

Organizations with established telehealth infrastructure prior to the COVID-19 pandemic were better positioned to rapidly expand services [94]. Emergency departments with prior telehealth experience noted that having established processes for training clinicians, existing relationships with technology vendors, stockpiles of hardware and peripherals, and established workflows facilitated implementation of new or expanded programs during the pandemic [94].

#### 3.5.3. Reimbursement and Policy

Reimbursement represents a critical factor affecting tele-nutrition sustainability, particularly in the United States healthcare context [11]. During the COVID-19 pandemic, temporary policy changes expanded coverage for telehealth services, including nutrition care provided by registered dietitians [95]. However, the permanence of these policies varies substantially by payer and jurisdiction [96]. As of 2024, analysis of U.S. state telehealth laws reveals significant variation in reimbursement approaches [97]. Interstate licensure barriers in the U.S. are being addressed through the development of professional healthcare compacts. The Dietitian Licensure Compact has been finalized and made available to states for enactment, though implementation remains in progress as the compact requires adoption by seven states to become active [98]. (For international perspectives, including Israeli implementation experience, see Section 3.7).

### 3.6. Barriers and Facilitators

#### 3.6.1. Access and Equity

Digital inequity represents a substantial barrier to equitable tele-nutrition access [13,30]. Families lacking reliable high-speed internet, appropriate devices, or digital literacy skills may be excluded from virtual care, with these barriers disproportionately affecting lower-income households, racial and ethnic minorities, older adults, and rural residents [99,100]. National survey data on telehealth utilization revealed that among telehealth users, the highest share of video-enabled visits occurred among those earning at least $100,000 (68.8%), while video telehealth rates were lowest among those without a high school diploma (38.1%), adults ages 65 and older (43.5%), Latino (50.7%), Asian (51.3%) and Black individuals (53.6%) [101].

Rural families face particular challenges with internet connectivity, with approximately 28% of people in rural areas lacking access to high-speed broadband internet compared to better connectivity in urban areas [102]. Rural residents experience more barriers to telehealth access than non-rural residents [99,100].

#### 3.6.2. Language and Cultural Considerations

Language access services are essential for equitable virtual care delivery. Professional interpretation services for telehealth visits via phone or video are increasingly available from multiple vendors. Successful implementation requires coordination, including seamless integration with telehealth platforms, training for clinicians on working with interpreters virtually, and ensuring platforms support multilingual content [103].

Cultural preferences regarding healthcare delivery vary and affect telehealth acceptability, with community engagement and culturally relevant design being important considerations [104]. Research demonstrates that those with prior telehealth experience are significantly more likely to prefer and feel comfortable with telehealth, with patients becoming more willing to use telemedicine as they gain experience and understanding of when it is appropriate [104]. Studies consistently show that experience with high-quality telehealth services leads to high satisfaction rates, with the majority of patients reporting positive experiences and finding telehealth to be an acceptable modality for receiving care [105].

#### 3.6.3. Physical Assessment Limitations

The inability to conduct in-person physical examinations remains a significant limitation of tele-nutrition, with providers relying on caregiver-obtained anthropometric measurements and being unable to perform comprehensive clinical assessments [106]. While caregivers can measure height and weight at home, studies examining parent-reported versus professionally measured anthropometrics found that most parents were reasonably accurate in reporting child height and weight, though body mass index calculated from parent-reported data had poor concordance with objectively measured data [107]. Research shows that diagnostic measures were more accurate when parents measured their child’s weight and height at home compared to when dimensions were based on parental estimations without measurement, with sensitivity for identifying overweight/obesity improving from 47% to 73% when parents actually measured rather than estimated [108].

More detailed assessments including mid-arm circumference, skinfold thickness, or body composition are not feasible remotely, with dietitians expressing concerns that the inability to palpate makes it harder to assess deficits in muscle and fat stores [109]. Physical examination for signs of nutritional deficiencies—including assessment of muscle and fat mass, appearance of skin, hair, eyes, fingernails, oral health, and signs of bone disease—requires in-person evaluation as part of a comprehensive nutrition-focused physical examination [110].

### 3.7. International Implementation: The Israeli Experience

Israel represents an example of rapid tele-nutrition adoption within a technologically advanced healthcare system. The country’s universal healthcare coverage through four competing health maintenance organizations (HMOs)–Clalit, Maccabi, Meuhedet, and Leumit—facilitated systematic telehealth integration during the COVID-19 pandemic.

Sheba Medical Center, ranked among the world’s top hospitals, established Sheba BEYOND in 2020 as Israel’s first virtual hospital, providing telemedicine services across multiple specialties including pediatrics and nutrition [111]. During the COVID-19 crisis, Sheba BEYOND provided over 50,000 virtual consultations to patients in Israel and internationally, with services including dietitian consultations as part of comprehensive virtual care (89). The virtual hospital model enables patients throughout Israel to access specialist services regardless of geographic location, addressing disparities between urban centers and peripheral regions.

Israeli implementation of tele-nutrition highlights several unique considerations relevant to diverse healthcare settings. The country’s multicultural population, including Jewish, Arab, Druze, and Bedouin communities, requires culturally adapted dietary counseling and multilingual service delivery across Hebrew, Arabic, Russian, and other languages. Geographic diversity, ranging from dense urban areas like Tel Aviv to rural communities in the Negev and Galilee regions, creates varying levels of technological infrastructure and internet connectivity affecting telehealth access [112,113].

The Israeli experience demonstrates that successful tele-nutrition implementation requires attention to cultural context, language accessibility, technological infrastructure variability, and healthcare system structure. While specific pediatric tele-nutrition outcome data from Israeli centers remain limited in the published literature, the rapid scaling of virtual nutrition services during the pandemic period provides insights into feasibility of large-scale telehealth integration within universal healthcare systems serving diverse populations [114].

Key barriers and facilitators to pediatric tele-nutrition implementation across diverse settings are systematically presented in Table 3.

**Table 3:** Barriers and Facilitators to Pediatric Tele-nutrition Implementation.

Domain [References]	Barriers	Facilitators	Strategies to Address Barriers	Geographic/Context Notes
**Technology and Infrastructure** [16,84,85,104]	• Poor internet connectivity • Lack of appropriate devices • Platform technical difficulties during sessions • Low digital literacy among caregivers • Technology fatigue, especially in children • Multiple platform requirements across providers	• High-speed broadband access • User-friendly, intuitive platforms • EHR integration reducing duplicate data entry • Technical support availability 24/7 • Multiple device compatibility (phone, tablet, computer) • Experience reduces technical difficulties over time	• Device lending programs for low-income families • Internet hotspots or connectivity subsidies • Audio-only visit options when video fails • Community technology hubs (libraries, clinics) • Comprehensive platform training with practice sessions • Prior experience improves comfort and reduces difficulties	Universal barrier but severity varies: • Rural areas face greater connectivity challenges than urban areas • LMIC settings: May require mobile-first, SMS-based platforms • HIC settings: Generally better infrastructure but disparities persist
**Clinical Assessment** [22,39,106,107,108,109,110]	• Cannot perform direct anthropometry (accuracy concerns with parent measurements) • Limited physical examination (cannot palpate edema, assess muscle/fat stores) • Cannot assess oral motor function for feeding disorders • Missing non-verbal cues in child behavior • No body composition assessment (skinfolds, bioimpedance) • Difficulty visualizing subtle signs of malnutrition	• Home environment observation provides contextual insights • Real-time feeding behavior observation in natural setting • Photo/video documentation between visits • Connected digital scales for objective weight data • Parent measurement training improves accuracy vs. estimation • Screen sharing enables real-time label review	• Hybrid models with periodic in-person visits for comprehensive assessment • Structured measurement training for parents (video demonstrations) • Clear protocols defining when in-person evaluation mandatory • Parents measuring (not estimating) improves accuracy: sensitivity 73% vs. 47% • Low threshold for transitioning to in-person when concerns arise • Validated assessment checklists for virtual encounters	Universal challenge across all settings • Protocols for hybrid care needed regardless of country • Connected devices more available in HIC but emerging in LMIC • Assessment limitations consistent globally
**Equity and Access** [13,31,32,86,94,101]	• Income-based disparities: 38.1% video use (income <$25 K) vs. 68.8% (≥ $100 K) • Race/ethnicity gaps: Latino 50.7%, Asian 51.3%, Black 53.6% vs. White populations • Education gaps: 38.1% (no HS diploma) • Language barriers without interpretation • Cultural preferences for in-person care • Digital literacy gaps across age/education • Privacy concerns in shared living spaces • Rural connectivity challenges	• Eliminates geographic travel barriers • Flexible scheduling accommodates work schedules • Reduced time burden (no commute, parking) • Access to distant specialists previously unavailable • Multiple family members can participate across locations • Reduced stigma for some conditions • Lower indirect costs (childcare, transportation)	• Professional interpretation services available via phone or video • Multilingual platform interfaces • Cultural adaptation of virtual care delivery • Proactive outreach to underserved populations • Subsidized technology access programs • Community partnerships for support • Monitor utilization by demographics to identify gaps • Maintain robust in-person options without creating two-tier system	Barrier severity varies by setting: • U.S.: Documented racial/ethnic/income disparities in utilization • Universal healthcare systems: May have different equity patterns • LMIC: Equity concerns more severe; require targeted interventions • Rural vs. urban: Greater connectivity gaps in rural areas
**Provider Factors** [20,89,90,91]	• Initial discomfort with virtual assessment techniques • Reimbursement uncertainty creating hesitancy • Technology difficulties and troubleshooting stress • Documentation uncertainty for virtual visits • Perceived limitations in building therapeutic relationship • Concerns about liability and quality of care • Training time requirements	• Increased scheduling flexibility • Reduced/eliminated commute for providers • Wider geographic reach to underserved areas • Home-based practice options • Confidence increased: 61% pre-pandemic to 76.6% during pandemic • Younger providers (20–39 yrs) adapt more readily • Peer learning and mentorship opportunities	• Comprehensive telehealth training programs (assessment techniques, communication) • Mentorship pairing experienced/novice providers • Clear documentation guidelines and templates • Ongoing technical support for providers • Quality metrics and feedback • Experience improves confidence: 78% RDN adoption rate during pandemic • Protected time for training and practice sessions	Relatively universal across settings • Training needs consistent globally • Younger providers adapt faster (universal finding) • Documentation requirements vary by country/system
**Organizational and System** [27,93,94,96,97]	• Variable reimbursement policies across payers • Interstate licensure restrictions (U.S.-specific) • Lack of institutional leadership support • Inadequate EHR integration with telehealth platforms • Workflow disruption during implementation • Start-up costs (technology, training) • Quality metrics not established for virtual care	• Strong leadership support and vision • Organizational prior telehealth experience • Robust IT infrastructure already in place • EHR-integrated telehealth platforms • COVID-19 policy changes enabled rapid scaling • Value-based payment models support telehealth • Champions within organization	• Advocacy for permanent telehealth reimbursement parity • Interstate licensure compacts (Dietitian Compact now available for state adoption) • Investment in digital infrastructure and support staff • Workflow optimization and process mapping • Quality metric development and tracking • Implementation science frameworks (CFIR) • Phased rollout with evaluation	Highly context-dependent: • U.S.: Interstate licensure major barrier; state-by-state reimbursement variation • Universal systems (Israel, Europe): Different regulatory structures; HMO coordination • LMIC: Infrastructure barriers more severe; different organizational models • COVID policy changes primarily HIC phenomenon
**Family Factors** [21,28,83,84,87,88]	• Initial preference for in-person care, especially first visits • Privacy concerns with virtual visits at home • Household disruptions (siblings, noise) • Young children difficulty maintaining focus (ages 0–5 lowest satisfaction) • Work schedule conflicts for live video • Caregiver stress operating technology • Preference for “hands-on” clinical experience	• Convenience and time savings highly valued • Reduced travel burden (cost, time, stress) • Home comfort reduces child anxiety • Ability to include working parents remotely • Adolescents value privacy and autonomy (73% prefer virtual) • High satisfaction reported after telehealth implementation • Observation of real home environment	• Hybrid models honoring family preferences • Flexible visit options (scheduled video, asynchronous, phone) • Clear privacy protocols and recommendations • Brief, focused virtual visits for young children (15–20 min) • Asynchronous options for busy families • Age-appropriate engagement strategies • Technical “dry runs” before first visit	Preferences vary culturally: • Some cultures stronger preference for in-person • Family structure affects participation (multi-generational homes) • Privacy concerns greater in crowded housing • Work flexibility varies by country/labor laws
**Reimbursement and Policy** [11,19,26,27,96,97,98,99]	• Inconsistent payer coverage for nutrition services • State-by-state variation in telehealth laws (U.S.) • Uncertainty of COVID-19 policy permanence • Nutrition services historically under-covered • Prior authorization requirements • Time limits on visits • Audio-only visits often not covered	• Temporary COVID-19 telehealth parity policies (U.S.) • Growing recognition of telehealth value • Value-based care models support prevention • Prevention focus in some policies • Cost-effectiveness potential (family savings documented) • Bipartisan policy support in some jurisdictions	• Advocacy for permanent parity policies • Documentation of cost-effectiveness and clinical outcomes • Demonstration projects showing value • Multi-stakeholder policy engagement • Professional organization lobbying (Academy of Nutrition and Dietetics) • Interstate compacts for licensure (7 states needed for activation) • Economic evaluation research	Highly country/system specific: • U.S.: Complex payer landscape; state laws vary; interstate practice restricted • Israel: Universal coverage via 4 HMOs; different barriers • Europe: Varies by country; many have universal systems • LMIC: Payment models completely different; may rely on government/NGO funding
**Measurement and Data Quality** [39,107,108]	• Parent-reported anthropometrics less accurate than professional • BMI from parent data: poor concordance • Growth velocity calculations may be inaccurate • Variability in home measurement technique • Delayed recognition of growth problems • Cannot obtain detailed body composition • Dietary recall accuracy concerns	• Connected digital scales can transmit weight data automatically • Photo food diaries provide visual documentation • Parents measuring vs. estimating: 73% vs. 47% sensitivity for obesity • Frequent home monitoring enables trend detection • Real-time data sharing with providers • Automated alerts for concerning values	• Detailed parent training in measurement technique (video demonstration + return demo) • Provide standardized measuring tools (tapes, stadiometers) • Connected scale programs for high-risk patients • Periodic in-person verification measurements • Heightened suspicion when measurements inconsistent with clinical picture • Clear protocols: when to require in-person assessment	Technology availability varies: • Connected scales more available/affordable in HIC • LMIC may rely on simple techniques • Training approaches adaptable globally • Verification frequency depends on resources

Note on References: Reference numbers correspond to supporting evidence in the manuscript. Specific statistics cited include: rural broadband access (28% lack access) [102], income-based video telehealth utilization disparities (38.1% for <$25 K vs. 68.8% for ≥$100 K) [101], race/ethnicity telehealth gaps [101], provider confidence changes (61% to 76.6%) [90], RDN telehealth adoption (78% during pandemic) [89], and parent measurement accuracy (73% vs. 47% sensitivity) [108]. Reimbursement and licensure barriers are primarily specific to the United States healthcare system, though payment models affect all settings.

## 4. Discussion

### 4.1. Principal Findings

This narrative review demonstrates that pediatric tele-nutrition represents a viable and effective care delivery model with clinical outcomes generally comparable to traditional in-person care across diverse populations and conditions. The evidence base has expanded substantially, with multiple randomized controlled trials and systematic reviews supporting tele-nutrition effectiveness.

Key findings include: (1) clinical outcomes for tele-nutrition are generally comparable to in-person care, with high-quality evidence from RCTs supporting non-inferiority for obesity and diabetes management; (2) patient and family satisfaction consistently exceeds 80% across most studies; (3) tele-nutrition is associated with significant benefits related to access, convenience, and reduced family burden; (4) hybrid models appear to optimally balance benefits and limitations; and (5) substantial barriers related to digital equity and reimbursement persist.

### 4.2. Technology as Enabler of Care Transformation

Technology serves not merely as a substitute delivery mechanism but as an enabler of care transformation. The ability to conduct nutrition assessments in families’ home environments provides contextual insights unavailable in clinical settings [18]. Increased frequency of contact enabled by virtual platforms may contribute to better outcomes through enhanced monitoring and continuous support [17]. Integration of remote monitoring devices, mobile health applications, continuous glucose monitors, and artificial intelligence algorithms creates opportunities for data-driven, personalized nutrition management [42,50]. However, technology also introduces challenges. The digital divide risks exacerbating health disparities if virtual care becomes predominant without addressing access barriers [13,31].

#### Mechanisms of Effectiveness

Understanding how pediatric tele-nutrition achieves outcomes comparable to in-person care requires consideration of several hypothesized mechanisms. First, virtual modalities may enable increased frequency of contact by reducing logistical barriers to visits. When families do not need to travel, take time off work, or arrange childcare, shorter and more frequent check-ins become feasible [17]. This enhanced monitoring may support better adherence and earlier identification of challenges.

Second, home environment observation provides contextual insights unavailable in clinical settings. Through video consultations, dietitians can observe actual family food environments, see what foods are available in the home, watch real-time feeding interactions, and identify contextual barriers to recommendations [18,56]. This authentic view of daily nutrition practices may lead to more tailored, realistic interventions.

Third, reduced participation barriers may improve engagement, particularly among families who would otherwise struggle to attend in-person visits. Elimination of travel time, transportation costs, parking fees, and time away from work or school reduces burden substantially [17]. For families managing children with chronic conditions requiring frequent nutrition visits, these barriers can be prohibitive.

Fourth, enhanced family involvement may occur when virtual platforms allow multiple caregivers to participate simultaneously from different locations [37]. Working parents who cannot attend daytime clinic appointments may join virtual visits from their workplace. Grandparents or other caregivers involved in food preparation can participate easily [37].

Finally, for adolescents, the privacy and autonomy afforded by virtual consultations from home may improve engagement compared to accompanying parents to clinical appointments. Studies suggest many adolescents prefer virtual care for its convenience and privacy [60,88]. These mechanisms likely vary in importance across different conditions, age groups, and family circumstances, contributing to heterogeneity in outcomes observed across studies. Understanding these pathways can inform optimization of tele-nutrition interventions and appropriate patient selection for virtual versus in-person care.

### 4.3. Optimal Care Delivery Models

Evidence suggests that hybrid models strategically combining virtual and in-person care may be optimal for many pediatric populations [32,52]. Initial comprehensive assessments, visits requiring extensive anthropometry or physical examination, and situations involving clinical concerns benefit from in-person evaluation. Subsequent follow-up visits, dietary counseling, education, and ongoing monitoring can often be delivered effectively via tele-nutrition.

The optimal ratio of virtual to in-person visits likely varies by condition, patient age, family preferences, and clinical stability. Flexibility to adjust care delivery modality based on changing clinical needs supports responsive, individualized care [23,33,40].

**Figure 2:**
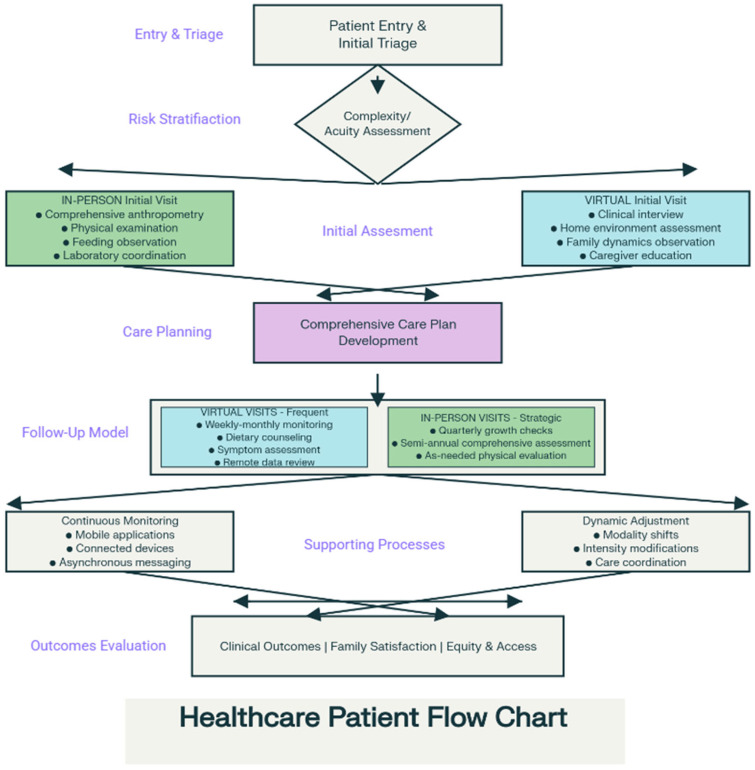
Conceptual Framework for Hybrid Pediatric Tele-Nutrition Care Model.

Figure 2 displays a suggested conceptual framework for hybrid Pediatric Tele-nutrition Care Model. This conceptual framework illustrates the hybrid pediatric tele-nutrition care model. Following initial triage and risk stratification, patients receive either in-person or virtual initial assessments based on clinical complexity. A comprehensive care plan guides hybrid follow-up combining frequent virtual visits for monitoring and counseling with strategic in-person visits for growth assessment and physical examination. Continuous monitoring through digital tools and dynamic care adjustment based on evolving needs support ongoing optimization. The model emphasizes flexible, family-centered care with systematic outcome evaluation across clinical, satisfaction, and equity domains.

### 4.4. Equity and Access Considerations

While tele-nutrition has potential to expand access, substantial equity concerns persist [13,31,93]. Digital inequity based on socioeconomic status, race/ethnicity, geography, and language may prevent vulnerable populations from benefiting. National survey data demonstrate that families with higher incomes and educational attainment utilize tele-nutrition at higher rates.

Specific disparities in telehealth access include: Video-enabled telehealth utilization was lowest among those earning less than $25,000 annually (38.1% video utilization) compared to 68.8% among those earning $100,000 or more [101]. Similarly, video telehealth rates were lower among those without a high school diploma (38.1%), adults ages 65 and older (43.5%), and among Latino (50.7%), Asian (51.3%), and Black individuals (53.6%) compared to White populations [101]. Rural families face particular connectivity challenges, with data indicating approximately 28% of people in rural areas lack access to high-speed broadband internet compared to substantially better connectivity in urban areas [102].

Addressing equity requires multifaceted approaches: ensuring technology access through device lending programs and internet subsidies; optimizing platforms for low bandwidth and audio-only options; maintaining robust in-person options for those who need or prefer them; providing technical support and interpretation services [103]; and designing culturally responsive virtual care experiences [40,93,104]. Healthcare systems must monitor tele-nutrition utilization and outcomes across demographic groups to identify and address disparities proactively.

### 4.5. Economic and Policy Considerations

Economic evidence for pediatric tele-nutrition remains limited and preliminary. While reductions in family-borne costs are well-documented—including travel expenses, parking fees, time away from work, and childcare costs [11,12]-comprehensive economic evaluations from a healthcare system perspective are lacking.

The hypothesis that tele-nutrition may be cost-effective requires rigorous evaluation incorporating multiple cost domains: initial implementation costs (technology platforms, equipment, staff training), ongoing operational costs (technical support, platform licensing, maintenance, security), provider time costs (which may differ from in-person care), and opportunity costs of transitioning care delivery models. Additionally, comprehensive analyses must account for potential cost savings from improved outcomes, reduced acute care utilization, and earlier intervention, while considering costs associated with maintaining parallel in-person and virtual infrastructure during transition periods.

Until such comprehensive cost-effectiveness analyses using established health economic frameworks (e.g., cost-utility analysis with quality-adjusted life years) are conducted across different pediatric populations and delivery models, cost-effectiveness claims remain speculative. Future research should prioritize economic evaluation alongside clinical effectiveness studies, particularly for conditions requiring intensive, long-term nutrition management where cumulative cost differences may be substantial.

Policy considerations 

It is important to note that reimbursement challenges and policy barriers described in this review are predominantly from the U.S. healthcare context, where fragmented payer systems and state-by-state regulations create complexity not present in countries with universal healthcare systems. International variations exist, with universal healthcare systems in countries like Israel and several European nations having different regulatory frameworks [113] and often smoother pathways to telehealth integration once organizational decisions are made. In the United States, reimbursement varies substantially across payers and jurisdictions [11,26,97]. Many temporary coverage expansions implemented during COVID-19 remain uncertain regarding permanence [96]. International variations exist, with universal healthcare systems in countries like Israel and several European nations having different regulatory frameworks [113]. Interstate practice regulations complicate care delivery across state lines, though professional licensure compacts are under development [98]. Advocacy for permanent telehealth parity in reimbursement continues, with particular challenges for nutrition services which have historically faced coverage limitations [19,96].

### 4.6. Future Directions

The field continues to evolve rapidly with several promising directions. Artificial intelligence applications may enhance efficiency through automated dietary assessment using image recognition technology [49], clinical decision support integrating multiple data streams [41], and personalized intervention recommendations based on individual response patterns [42]. Precision nutrition approaches incorporating genetic, microbiome, and metabolomic data may enable increasingly individualized dietary recommendations tailored to individual biological characteristics [42,50].

At a population health level, tele-nutrition could enable large-scale preventive interventions and early identification of at-risk children through integration with electronic health records and population health management platforms. School-based virtual nutrition programs could reach children who would not otherwise access specialized nutrition services.

Applications in Low- and Middle-Income Settings

Nearly all included studies (>95%) were conducted in high-income countries with advanced telecommunications infrastructure, primarily the United States, Canada, Europe, Australia, and Israel. The applicability of these findings to low- and middle-income settings where the global burden of pediatric malnutrition is highest remains uncertain [3].

LMIC contexts present distinct challenges and opportunities. Infrastructure limitations may necessitate different technological approaches—mobile-first platforms rather than computer-based systems, SMS-based interventions rather than video consultations, and integration with existing community health worker networks rather than specialist dietitian services [35]. The predominance of undernutrition rather than overnutrition in many LMICs requires different assessment focus and intervention strategies compared to the obesity and chronic disease management that dominates HIC tele-nutrition literature.

However, hub-and-spoke models connecting specialized pediatric nutrition expertise at tertiary centers with frontline community health workers in peripheral areas show promise [89]. Task-shifting approaches where community health workers deliver nutrition interventions with remote specialist supervision and training may extend limited specialist capacity. Mobile health applications for growth monitoring, feeding education, and micronutrient supplementation reminders could reach populations with limited healthcare access.

Successful telehealth models from other specialties in LMIC contexts demonstrate feasibility despite infrastructure challenges. Future research and implementation efforts should prioritize adaptation of tele-nutrition approaches to resource-limited settings where need is greatest. Partnerships between institutions in high-income countries and those in LMICs could facilitate knowledge transfer and capacity building while ensuring cultural appropriateness and local relevance.

### 4.7. Limitations and Research Priorities

While the evidence base has expanded substantially, important limitations remain. Many studies have relatively short follow-up periods, limiting understanding of long-term sustainability and outcomes. Certain populations remain underrepresented including very young children (particularly infants), those with developmental disabilities affecting communication, and non-English speaking families.

Most telehealth studies report on general pediatric or multi-specialty services rather than nutrition-specific encounters, limiting our ability to provide precise utilization statistics specifically for pediatric tele-nutrition.

The geographic concentration of studies in high-income countries with advanced telecommunications infrastructure limits generalizability to low- and middle-income settings where pediatric malnutrition burden is highest [3]. Different technological approaches (e.g., SMS-based platforms, integration with community health workers) may be more appropriate in resource-limited settings, but evidence remains limited.

Most included studies did not report outcomes stratified by socioeconomic status, race/ethnicity, language, or other equity-relevant characteristics, limiting our ability to assess differential effectiveness across populations. This represents a critical gap given concerns about digital inequity potentially exacerbating existing health disparities [13,31,101].

The evidence base varies in quality across populations and conditions. While robust randomized controlled trial evidence supports tele-nutrition effectiveness for pediatric obesity and diabetes management, other conditions such as feeding disorders, metabolic diseases, and preventive nutrition rely more heavily on observational studies, case series, and implementation reports. Where we cite meta-analyses or pooled effect estimates, we have specified the source, methods, and sample size, but readers should note that much of the literature consists of single-site observational studies with moderate sample sizes.

Research priorities include:

1. Long-term outcome studies examining sustained effects over multiple years, including growth trajectories, disease progression, and quality of life
2. Comparative effectiveness research comparing different tele-nutrition modalities (synchronous video vs. asynchronous vs. hybrid) and implementation strategies
3. Implementation science investigating effective integration strategies, organizational factors supporting adoption, and sustainment over time
4. Health equity research examining barriers and facilitators across diverse populations, with outcomes stratified by socioeconomic status, race/ethnicity, language, geography, and other relevant characteristics
5. Economic evaluations including comprehensive cost-effectiveness analyses from multiple perspectives (family, payer, healthcare system, societal)
6. Child and adolescent perspectives through age-appropriate qualitative research understanding preferences, experiences, and developmental considerations
7. Provider competency development studying optimal training approaches, assessment techniques adapted for virtual environments, and quality metrics
8. LMIC adaptation studies examining feasibility, acceptability, and effectiveness of tele-nutrition models in resource-limited settings

### 4.8. Risks and Safeguards

While this review emphasizes the benefits and effectiveness of pediatric tele-nutrition, clinicians and healthcare systems must recognize and mitigate important risks inherent to virtual care delivery.

Clinical Assessment Risks

The inability to perform hands-on physical examination creates risk of missed clinical findings. Subtle signs of malnutrition—including loss of subcutaneous fat, muscle wasting, skin changes, oral health problems, or edema—may not be apparent through video consultation [110]. Oral motor function assessment for feeding disorders is substantially limited without direct observation of oral structures and swallowing mechanics [9]. Growth trajectory concerns may be delayed if anthropometric measurements are inaccurate or infrequent [22,39].

Safeguards include establishing clear protocols defining when in-person evaluation is mandatory (e.g., new patients with complex conditions, clinical deterioration, growth concerns), maintaining a low threshold for transitioning to in-person care when clinical concerns arise, using structured assessment checklists to systematically review for warning signs requiring in-person evaluation, and implementing hybrid models with periodic in-person visits for comprehensive physical assessment [32,52].

Technology-Related Risks

Screen fatigue, particularly in young children, may limit engagement and assessment quality during prolonged video consultations [87]. Platform technical failures during critical consultations can disrupt care continuity and create frustration for families already struggling with child nutrition concerns. Inadequate internet connectivity may result in incomplete assessments, misunderstandings of dietary recommendations, or inability to observe feeding behaviors adequately [84,85]. Audio-only consultations when video fails provide substantially limited clinical information, particularly for pediatric populations where visual assessment of child appearance, feeding skills, and family interactions is important [16].

Safeguards include limiting session lengths for young children (15–20 min for preschoolers, 30–40 min for school-age children), having backup communication plans with phone numbers readily available and pre-tested, ensuring 24/7 technical support availability for both families and providers, conducting platform tests before appointments with new families, providing clear instructions and practice sessions for families unfamiliar with technology, and being prepared to reschedule rather than conduct inadequate assessments with poor connectivity [93,94].

Privacy and Security Concerns

Ensuring HIPAA compliance and data security in home-based consultations presents unique challenges. Confidentiality may be compromised when other household members are present and can overhear sensitive discussions about eating behaviors, weight concerns, or family dynamics [21]. Adolescents may be unable to speak freely about eating concerns, body image issues, or mental health symptoms if parents or siblings are nearby. Unsecured home internet networks or use of non-encrypted platforms create data breach risks and potential exposure of protected health information.

Safeguards include using only secure, encrypted, HIPAA-compliant telehealth platforms approved by institutional security teams [19], recommending private spaces for consultations when possible and providing guidance on how families can create privacy (e.g., using headphones, closing doors), establishing clear consent processes addressing privacy limitations in home environments and documenting family preferences, offering phone-only options for sensitive discussions where adolescents or parents need confidential conversation, and providing clear guidance on platform security to families including avoiding public Wi-Fi networks [103].

Equity and Access Risks

If virtual care becomes the predominant modality without addressing access barriers, existing health disparities may be exacerbated rather than reduced [13,31]. Families lacking reliable internet, appropriate devices (smartphones may be inadequate for quality video), or digital literacy skills may be excluded from care or receive inferior quality services. Language barriers may be more difficult to address virtually if interpretation services are not seamlessly integrated into telehealth platforms [103]. Older caregivers or those with limited education may struggle with technology navigation even when devices and connectivity are available.

Safeguards include maintaining robust in-person care options for those who need or prefer them without creating two-tiered care systems, implementing device lending programs providing tablets or laptops to families in need, providing internet hotspots or subsidies for families with connectivity barriers, offering technology navigation assistance in multiple languages through dedicated support staff, conducting outreach to underserved populations to proactively offer support, and monitoring utilization patterns across demographic groups to identify and address emerging disparities before they become entrenched [40,93,99,100].

Provider Competency Risks

Dietitians inadequately trained in virtual assessment techniques may provide suboptimal care or miss important clinical information. Overreliance on patient-reported information without strategies to verify accuracy creates risk of inappropriate interventions based on incomplete or inaccurate data. Inability to perform typical assessment techniques (palpation of edema, detailed anthropometry including skinfold measurements, observation of oral motor skills) may lead to misdiagnosis or delayed recognition of problems [109]. Providers uncomfortable with technology may experience increased stress and reduced clinical effectiveness.

Safeguards include comprehensive telehealth training programs covering modified assessment techniques adapted for virtual environments, communication strategies for building rapport through video, and troubleshooting common technology problems, competency assessments before independent tele-nutrition practice ensuring providers can conduct thorough virtual assessments, ongoing mentorship and peer learning opportunities with experienced tele-nutrition providers, clear documentation standards for virtual encounters specifying what was assessed and what limitations existed, and regular quality audits of tele-nutrition care through chart review and patient feedback [20,89,90].

Measurement Accuracy Risks

Reliance on caregiver-reported anthropometric measurements introduces accuracy concerns that could affect clinical decision-making. Studies show parent-measured height and weight are more accurate than estimates, but still show variability compared to professional measurements [107,108]. Body mass index calculated from parent-reported data may have poor concordance with objectively measured data, potentially leading to misclassification of nutritional status [107]. Growth velocity calculations based on inaccurate measurements could lead to inappropriate interventions or delayed recognition of growth problems.

Safeguards include providing families with detailed measurement training, ideally with video demonstration and return demonstration to verify technique, supplying standardized measurement tools when possible (e.g., measuring tapes, growth charts) to reduce variability, using connected digital scales that transmit weight data directly to healthcare providers to avoid transcription errors and enable frequent monitoring, conducting periodic in-person verification measurements to calibrate home measurements and identify systematic errors, and maintaining heightened clinical suspicion when measurements seem inconsistent with reported symptoms or clinical trajectory, with low threshold for in-person evaluation [39,72].

Healthcare organizations implementing pediatric tele-nutrition programs must develop comprehensive risk mitigation strategies addressing these domains. Quality assurance processes should include systematic monitoring for adverse events, near-misses, and quality concerns specific to virtual care delivery. Regular review of cases where virtual care led to delays in diagnosis or treatment can inform protocol improvements. Family and provider feedback mechanisms should specifically elicit concerns about virtual care quality and safety.

### 4.9. Implications for Practice and Policy

For practitioners, evidence-based recommendations include: implementing clear criteria for virtual versus in-person care based on clinical complexity, assessment needs, and family circumstances; considering hybrid models for most populations balancing convenience with comprehensive assessment; investing in comprehensive telehealth training covering both technical skills and modified clinical assessment techniques; selecting reliable, user-friendly platforms that work across multiple devices and internet speeds; proactively addressing digital equity through technology support and maintaining in-person options; providing clear family preparation including platform testing and measurement training; implementing quality monitoring through patient feedback and outcome tracking; establishing care coordination processes ensuring smooth transitions between virtual and in-person care; and documenting virtual encounters appropriately including assessment limitations.

For policymakers and health system leaders, priorities include: establishing permanent reimbursement parity for tele-nutrition services across payers; supporting interstate licensure compacts enabling practice across state lines; investing in broadband infrastructure particularly in rural and underserved areas; funding digital inclusion programs providing devices and connectivity support; developing quality standards and performance metrics specific to tele-nutrition; prioritizing research funding for effectiveness, implementation, and equity studies; supporting workforce training initiatives in telehealth competencies; and providing integration incentives encouraging hybrid models rather than exclusively virtual or in-person approaches.

## 5. Conclusions

Pediatric tele-nutrition services have emerged as an effective care delivery model offering significant advantages for access, convenience, and family-centered care while achieving clinical outcomes comparable to traditional in-person services across multiple conditions and populations. The evidence base, though concentrated in high-income countries, includes robust randomized controlled trials and systematic reviews supporting effectiveness for obesity and diabetes management, with growing evidence for other pediatric nutrition conditions.

Benefits are particularly pronounced for families in rural or underserved areas where specialist access is limited, those managing chronic conditions requiring frequent monitoring where travel burden is prohibitive, and families facing logistical barriers to in-person care including work schedules, transportation challenges, or childcare constraints.

However, realizing the full potential of tele-nutrition requires addressing persistent challenges related to digital equity, with substantial disparities in access based on income, race/ethnicity, geography, and language; regulatory frameworks including reimbursement policies and interstate practice restrictions; physical assessment limitations requiring clear protocols for when in-person evaluation is essential; provider training needs in virtual assessment techniques and technology use; and mitigation of clinical, technological, privacy, and equity risks inherent to virtual care delivery.

Hybrid models thoughtfully combining virtual and in-person care appear optimal for many pediatric populations, leveraging the strengths of each modality (accessibility and convenience of virtual care; comprehensive physical assessment of in-person care) while mitigating limitations. The optimal balance varies by condition, age, clinical stability, and family circumstances, requiring flexible, individualized approaches.

As the field continues to mature, priorities include the following: expanding the evidence base through long-term outcome studies, comparative effectiveness research, and studies in underrepresented populations and settings; developing equity-promoting strategies ensuring tele-nutrition expands rather than restricts access for vulnerable populations; establishing sustainable reimbursement frameworks supporting appropriate use without creating financial barriers; optimizing technology integration including AI applications and precision nutrition approaches while maintaining focus on clinical relationships; strengthening quality and safety systems specific to virtual care delivery; and ensuring tele-nutrition remains grounded in child- and family-centered principles prioritizing individual needs and preferences.

The COVID-19 pandemic accelerated tele-nutrition adoption and demonstrated both feasibility and challenges at scale. As healthcare systems determine post-pandemic care delivery models, the opportunity exists to thoughtfully integrate tele-nutrition into comprehensive pediatric nutrition services in ways that maximize benefits for children and families while addressing limitations and equity concerns. With continued attention to evidence generation, implementation science, policy development, and quality improvement, pediatric tele-nutrition can fulfill its promise of improving nutritional care access and outcomes for all children, regardless of geographic location, socioeconomic status, or other barriers to traditional care.

## Figures and Tables

**Table 1:** Comparison of Pediatric Tele-nutrition Technology Platforms.

Platform Type	Adoption Prevalence	Key Advantages	Primary Limitations	Best Applications
Synchronous Video	85–95% of programs	• Real-time interaction • Visual assessment of feeding • Home environment observation • Immediate feedback • Screen sharing for education	• Requires scheduled time • Technology/bandwidth dependent • Less flexible for busy families • Digital literacy needed	• Initial assessments • Feeding disorder observation • Parent education • Complex diet counseling • Family engagement
Asynchronous Communication	30–45% of programs	• High scheduling flexibility • Reduced time burden • Ongoing monitoring between visits • Photo/video documentation • Convenient for families	• Delayed responses • Limited interaction depth • May miss urgent issues • Less personal connection	• Diet diary review • Label reading questions • Symptom tracking • Follow-up clarifications • Routine monitoring
Mobile Applications	45–65% of programs	• Continuous tracking • Automated reminders • Growth chart integration • Gamification for children • AI-enabled food recognition	• Requires digital literacy • Adherence varies • Data privacy concerns • Platform fragmentation • Cost barriers	• Diabetes management (CGM integration) • Food allergy tracking • PKU diet monitoring • Obesity self-monitoring • Medication reminders
Remote Monitoring Devices	20–35% of programs	• Objective data collection • Early problem detection • Reduced reporting burden • Real-time data sharing • Automated alerts	• Device cost • Technical setup required • Accuracy variability • Limited parameters • Requires broadband connectivity	• Failure to thrive (connected scales) • Growth monitoring • Diabetes (CGM) • Weight tracking • Early intervention
Hybrid Models	40–55% of programs	• Combines strengths of each modality • Flexibility based on clinical needs • Comprehensive assessment capability • Optimized resource use • Patient preference accommodation	• Complex coordination • Variable insurance coverage • Requires clear protocols • Higher organizational demands	• Most chronic conditions • Complex medical needs • Initial in-person + virtual follow-up • Periodic growth assessments

**Note:** Adoption rates represent the percentage of pediatric tele-nutrition programs utilizing each platform type. Categories are not mutually exclusive; most programs employ multiple modalities simultaneously (e.g., a program may use both synchronous video and mobile applications). Rates are derived from studies by Sauers-Ford et al. n = 347), Curfman et al. [44] (n = 892), Smith et al. (n = 215), Turner et al. (n = 658), Martinez et al. (n = 1768), and Krasovs.ky et al. (n = 351), representing a total of 4231 pediatric tele-nutrition encounters across diverse settings and conditions.

## Data Availability

The original data presented in the study are openly available in PubMed, CINAHL, Embase, and Web of Science databases.

## Supplementary Materials

The following supporting information can be downloaded at https://www.mdpi.com/article/10.3390/healthcare13233107/s1, **Supplementary Table S1.** Characteristics of All Included Studies (n = 114) [See separate detailed table artifact]. Available as online appendix with sortable columns for: Author/Year, Country, Study Design, Sample Size, Age Range, Condition/Population, Intervention Type, Key Outcomes, Evidence Level.

## Abbreviations

ARFID	Avoidant/Restrictive Food Intake Disorder
BMI	Body Mass Index
CGM	Continuous Glucose Monitor
CKD	Chronic Kidney Disease
EHR	Electronic Health Record
EoE	Eosinophilic Esophagitis
HbA1c	Hemoglobin A1c
HIC	High-Income Country
HMO	Health Maintenance Organization
IBD	Inflammatory Bowel Disease
LMIC	Low- and Middle-Income Country
PKU	Phenylketonuria
PTN	Pediatric Tele-Nutrition
RCT	Randomized Controlled Trial
RDN	Registered Dietitian Nutritionist
WIC	Women, Infants, and Children (nutrition program)
