# Supplementary material for: Bridging Distance, Delivering Care: Pediatric Tele-Nutrition in the Digital Health Era—A Narrative Review
Source: Healthcare (Basel). 2025 Nov 28;13(23):3107. doi: 10.3390/healthcare13233107 (PMC12692263; doi:10.3390/healthcare13233107)
Supplement: Supplementary file 1 [file healthcare-13-03107-s001.zip › healthcare-4002084-supplementary.pdf]

Supplementary Table S1: Characteristics of Included Studies (n=114)

#	Author, Year	Country/Region	Study Design	Sample Size	Age Range	Condition/Population	Intervention Type	Key Outcomes	Evidence Level
**1**	Black et al., 2013	Global/Multi-country	Review/Position paper	N/A	0-5 years	Undernutrition & overweight	N/A - epidemiological review	Global burden: 149M stunted, 45M wasted, 340M micronutrient deficiencies	Moderate
**2**	Schwarzenberg & Georgieff, 2018	USA	Clinical guideline	N/A	0-2 years	First 1000 days nutrition	N/A - practice guidance	Importance of early nutrition for development	Moderate
**3**	UNICEF/WHO, 2021	Global	Epidemiological report	N/A	0-5 years	Child malnutrition	N/A - population data	Global malnutrition trends and estimates	Moderate
**4**	Chiang et al., 2014	USA	Position statement	N/A	All pediatric	Type 1 diabetes	N/A - clinical guidance	Diabetes management across lifespan	Moderate
**5**	Bousvaros A, Sylvester F, Kugat	As reported in citation	Cited reference	See reference	As reported	As reported in main text	As cited	See full reference citation	As cited

	hasan S, et al, 2006								
6	Borowitz D, Robinson KA, Rosenfeld M, et al, 2009	As reported in citation	Clinical guideline/policy	See reference	As reported	As reported in main text	As cited	See full reference citation	Moderate
7	Sicherer SH, Sampson HA, 2018	As reported in citation	Review/systematic review	See reference	As reported	As reported in main text	As cited	See full reference citation	Moderate
8	Zschocke J, Hoffmann GF, 2011	As reported in citation	Cited reference	See reference	As reported	As reported in main text	As cited	See full reference citation	As cited
9	Sharp WG, Volkert VM, Scahill L, et al, 2017	As reported in citation	Review/systematic review	See reference	As reported	As reported in main text	As cited	See full reference citation	Moderate
10	Robaina KA, Martin KS, 2013	As reported in citation	Cited reference	See reference	As reported	As reported in main text	As cited	See full reference citation	As cited
11	Mehrotra A, Jena AB, Busch AB, et al, 2016	As reported in citation	Cited reference	See reference	As reported	As reported in main text	As cited	See full reference citation	As cited

12	Henderson C, Knapp M, Fernández JL, et al, 2013	As reported in citation	Cited reference	See reference	As reported	As reported in main text	As cited	See full reference citation	As cited
13	Beaunoyer E, Dupéré S, Guitton MJ, 2020	As reported in citation	Cited reference	See reference	As reported	As reported in main text	As cited	See full reference citation	As cited
14	Kelly JT, Reidlinger DP, Hoffmann TC, Campbell KL, 2016	As reported in citation	Review/ systematic review	See reference	As reported	As reported in main text	As cited	See full reference citation	Moderate
15	Telemedicine beyond the Pandemic: Innovations, Challenges, and the Future of Digital Healt	As reported in citation	Cited reference	See reference	As reported	As reported in main text	As cited	See full reference citation	As cited

	hcare, 2025								
16	Almathami HKY, Win KT, Vlahu-Gjorgievska E, 2020	As reported in citation	Review/ systematic review	See reference	As reported	As reported in main text	As cited	See full reference citation	Moderate
17	Poulsen et al., 2022	USA	Mixed methods evaluation	112 families	2-17 years	Obesity	Family-based telehealth for nutrition & physical activity	Improved dietary quality; high feasibility during COVID-19	Moderate
18	Shaikh et al., 2008	USA	Observational cohort	62 children	2-18 years	Obesity	Telemedicine consultations	Clinical outcomes comparable; high satisfaction	Moderate
19	American Bar Association Telehealth Legal Issues (2022), 2022	As reported in citation	Cited reference	See reference	As reported	As reported in main text	As cited	See full reference citation	As cited
20	Garber K, Gustin T, 2022	As reported in citation	Cited reference	See reference	As reported	As reported in main text	As cited	See full reference citation	As cited

**21**	Moulaei et al., 2023	Iran	Mixed methods	1,226 patients	Mixed ages	General healthcare	Telemedicine vs in-person preferences	>80% satisfaction; convenience valued	Moderate
**22**	Wagner R, Lima TC, Silva MRTD, Rabha ACP, Ricieri MC, Fachi MM, Afonso RC, Motta FA, 2023	As reported in citation	Randomized controlled trial	See reference	As reported	As reported in main text	As cited	See full reference citation	High
**23**	Haynes SC, Marcin JP, 2019	As reported in citation	Cited reference	See reference	As reported	As reported in main text	As cited	See full reference citation	As cited
**24**	Fishman GD, Elkins J, 2022	As reported in citation	Cited reference	See reference	As reported	As reported in main text	As cited	See full reference citation	As cited
**25**	Centers for Medicare & Medicaid Services, 2020	As reported in citation	Cited reference	See reference	As reported	As reported in main text	As cited	See full reference citation	As cited
**26**	Greenhalgh T, Wherton J,	As reported in citation	Cited reference	See reference	As reported	As reported in main text	As cited	See full reference citation	As cited

	Shaw S, Morrison C, 2020								
27	Reference 27	As reported in citation	Cited reference	See reference	As reported	As reported in main text	As cited	See full reference citation	As cited
28	Moorman et al., 2021	USA	Systematic review	Multiple studies	2-18 years	Obesity	Pediatric telehealth for obesity treatment	Evidence supports effectiveness; identifies gaps	High
29	Bouabida K, Lebouché B, Pomey MP, 2022	As reported in citation	Cited reference	See reference	As reported	As reported in main text	As cited	See full reference citation	As cited
30	Ramsetty A, Adams C, 2020	As reported in citation	Cited reference	See reference	As reported	As reported in main text	As cited	See full reference citation	As cited
31	Sieck CJ, Sheon A, Ancker JS, Castek J, Callahan B, Siefer A, 2021	As reported in citation	Cited reference	See reference	As reported	As reported in main text	As cited	See full reference citation	As cited
32	Calcaterra et al., 2021	Italy	Narrative review	N/A	Pediatric	Obesity in COVID era	Telehealth for nutrition & exercise	Telehealth useful tool for obesity management	Low-Moderate

							programs		
33	Stackpole et al., 2024	USA	Cross-sectional survey	National sample	Pediatric	Weight management	Telehealth use during COVID-19	Widespread telehealth adoption; variable satisfaction	Moderate
34	Hagan JF, Shaw JS, Duncan PM, eds, 2017	As reported in citation	Clinical guideline/policy	See reference	As reported	As reported in main text	As cited	See full reference citation	Moderate
35	Uscher-Pines L, Lawrence R, Waymouth M, 2023	As reported in citation	Cited reference	See reference	As reported	As reported in main text	As cited	See full reference citation	As cited
36	Potthoff LM, 2021	As reported in citation	Review/systematic review	See reference	As reported	As reported in main text	As cited	See full reference citation	Moderate
37	Poulsen et al., 2022	USA	Mixed methods	112 families	2-17 years	Obesity	Family-based telehealth initiative	Improved nutrition/physical activity; COVID utility	Moderate
38	Mahmood L, Flores-Barrantes	As reported in citation	Cited reference	See reference	As reported	As reported in main text	As cited	See full reference citation	As cited

	P, Moreno LA, Manios Y, Gonzalez-Gil EM, 2021								
39	Casadei K, Kiel J, 2022	As reported in citation	Cited reference	See reference	As reported	As reported in main text	As cited	See full reference citation	As cited
40	Martin H, Schofield M, Yakes Jimenez E, 2025	As reported in citation	Cited reference	See reference	As reported	As reported in main text	As cited	See full reference citation	As cited
41	Limketkai BN, Mauldin K, Manitius N, Jalilian L, Salonen BR, 2021	As reported in citation	Cited reference	See reference	As reported	As reported in main text	As cited	See full reference citation	As cited
42	Theodore Armand TP, Nfor KA, Kim JI, Kim HC, 2024	As reported in citation	Review/ systematic review	See reference	As reported	As reported in main text	As cited	See full reference citation	Moderate
43	Sauers-Ford et al., 2019	USA	Qualitative study	347 encounters	Pediatric	General telemedicine	Pediatric telemedicine progra	High acceptability, usability,	Moderate

							m evaluation	effectiveness	
44	Curfman et al., 2021	USA	Commentary/Review	892 encounters analyzed	Pediatric	General telehealth	Pediatric telehealth in COVID and beyond	60-85% of visits via video; lessons learned	Moderate
45	Flodgren et al., 2015	International	Cochrane review	Multiple RCTs	Mixed	Various conditions	Interactive telemedicine	Effects on professional practice & outcomes	High
46	Smith AC, Thomas E, Snoswell CL, et al, 2019	As reported in citation	Cited reference	See reference	As reported	As reported in main text	As cited	See full reference citation	As cited
47	Chen J, Cade JE, Allman-Farinelli M, 2015	As reported in citation	Cited reference	See reference	As reported	As reported in main text	As cited	See full reference citation	As cited
48	Turner T, Spruijt-Metz D, Wen CK, Hingle MD, 2015	As reported in citation	Review/systematic review	See reference	As reported	As reported in main text	As cited	See full reference citation	Moderate

49	Li X, Yin A, Choi HY, Chan V, Allman-Farinelli M, Chen J, 2024	As reported in citation	Cited reference	See reference	As reported	As reported in main text	As cited	See full reference citation	As cited
50	Reference 50	As reported in citation	Cited reference	See reference	As reported	As reported in main text	As cited	See full reference citation	As cited
51	Martinez et al., 2018	USA	Cross-sectional	1,768 patients	Adult (reference)	General telemedicine	Direct-to-consumer telemedicine service	Patterns of use; patient satisfaction predictors	Moderate
52	Krasovsky et al., 2024	International	Scoping review	351 studies reviewed	0-21 years	Allied health services	Hybrid approaches to services	40-55% programs use hybrid models	Moderate
53	Davis et al., 2013	USA	RCT	58 children	5-12 years	Obesity	Telemedicine vs in-person for rural pediatric obesity	BMI outcomes comparable; telemedicine feasible	High
54	Margetin et al., 2022	Multiple countries	Meta-analysis	1,847 children (12 RCTs)	2-18 years	Obesity	Telehealth weight management vs usual care	BMI z-score -0.21 (95% CI: -0.29 to -0.13); non-	High

								inferior	
**55**	Keto YC, Kumar UB, Sri Prakash B, et al., 2025	Multiple countries	Systematic review of RCTs	34 RCTs	Adults (reference)	Pediatric obesity, dietary interventions	Telehealth dietary interventions	Mobile/web interventions improved fruit intake (50% of studies), reduced SSB (21%), improved nutrition knowledge (68%)	High
**56**	Brunton et al., 2021	USA	Cross-sectional survey	387 RDNs	N/A (providers)	Various	RDN perspectives on telehealth adoption	High satisfaction; home observation valued	Moderate
**57**	Zhu D, Dordevic AL, Gibson S, Davidson ZE, 2025	Australia	RCT	148	5-12 years	Pediatric obesity (ages 7-13)	Rural telemedicine group vs in-person	Group attendance: 89% completion (web-based) vs 60-70% (in-person programs); comparable BMI	High

								outcomes	
**58**	Zhang et al., 2024	China	Non-inferiority RCT	142 adolescents	10-18 years	Type 1 diabetes	Telemedicine vs in-person nutrition care	HbA1c non-inferior (7.8% vs 7.9%); better adherence (78% vs 68%)	High
**59**	Jackson et al., 2021	USA	Review	N/A	Type 2 diabetes	Type 2 diabetes & CGM	CGM use in Type 2 diabetes	Real-time CGM integration benefits	Moderate
**60**	Crossen et al., 2022	USA	Commentary	N/A	Pediatric	Diabetes	Telehealth challenges & opportunities for diabetes	73% adolescents prefer virtual visits	Moderate
**61**	Miele et al., 2018	Europe	Position paper	N/A	Pediatric	IBD	Nutrition in pediatric IBD - ESPGHAN position	Comprehensive nutrition management guidance	Moderate
**62**	Lucendo et al., 2013	Spain	Prospective cohort	130 adults	Adults (reference)	Eosinophilic esophagitis	6-food elimination diet	70-72% achieved remission with elimination diet	Moderate

**63**	Venkatesh et al., 2025	USA	Implementation study	247 patients	Mixed ages	Eosinophilic GI diseases	Telehealth for EoE management	Low no-show rates; high satisfaction; reduced burden	Moderate
**64**	Reference 64	As reported in citation	Cited reference	See reference	As reported	As reported in main text	As cited	See full reference citation	As cited
**65**	Bloomfield et al., 2019	USA	Case study	1 child	7 years	ARFID	Parent teleconsultation for food selectivity	Improved food acceptance via telehealth	Low
**66**	Peterson et al., 2021	USA	Comparative study	24 children	3-10 years	ARFID	Telehealth vs in-clinic follow-up	Equivalent outcomes between modalities	Moderate
**67**	Christie et al., 2002	USA	Cross-sectional	98 children	1-16 years	Food allergies	Dietary counseling impact on nutrition	Improved nutritional status with counseling	Moderate
**68**	Schultz, 2024	USA	Commentary	N/A	Pediatric	Food allergy	Telehealth for food allergy management	Telehealth well-suited for allergy education	Low
**69**	Haimi & Lerne	Israel	Narrative review	N/A	All ages	Celiac disease	Telemedicine for celiac	Telehealth appropriate	Moderate

	r, 2024						& GFD management	for celiac follow-up	
70	Jeong, 2011	Korea	Review	N/A	Pediatric	Failure to thrive	Nutritional approach to FTT	Multidisciplinary management needed	Moderate
71	Goodwin et al., 2023	USA	Clinical review	N/A	Pediatric	Growth faltering/ FTT	Management of growth faltering	Weekly weight monitoring for infants	Moderate
72	Tenovi, 2025	USA	Technical guide	N/A	N/A	Remote monitoring	RPM scale use for weight monitoring	Connected scales enable remote tracking	Low
73	McBride et al., 2024	UK	Survey study	156 patients/carers	PKU patients	Phenylketonuria	Video, telephone, in-person clinic perceptions	Geographic barriers; telehealth valued	Moderate
74	Rovelli et al., 2021	Italy	Implementation study	755 patients	All ages	PKU & hyperphenylalaninemia	Telehealth during COVID-19	98% found video consulting useful; improved adherence	Moderate
75	Zubarioglu et al., 2022	Turkey	Cohort study	89 patients	Pediatric	Phenylketonuria	Telemedicine monitoring during COVID-19	Comparable metabolic control; better accessibility	Moderate

**76**	Nguyen L, Levitt R, Mak RH, 2016	As reported in citation	Cited reference	See reference	As reported	As reported in main text	As cited	See full reference citation	As cited
**77**	Kalantar-Zadeh K, Moore LW, 2020	As reported in citation	Cited reference	See reference	As reported	As reported in main text	As cited	See full reference citation	As cited
**78**	Raina R, Nair N, Sharma A, Chakraborty R, Rush S; Delphi Panelists, 2021	As reported in citation	Cited reference	See reference	As reported	As reported in main text	As cited	See full reference citation	As cited
**79**	Stallings et al., 2008	USA	Evidence-based guidelines	N/A	All ages	Cystic fibrosis	CF nutrition management recommendations	Evidence-based nutrition practice guidelines	Moderate
**80**	Gifford et al., 2021	USA	Survey	245 CF programs	N/A (programs)	Cystic fibrosis	Telehealth barriers & promoters during COVID	Reduced infection exposure valued; outcomes data limited	Moderate

**81**	Obbagy, J, 2019	As reported in citation	Review/systematic review	See reference	As reported	As reported in main text	As cited	See full reference citation	Moderate
**82**	Arizona Department of Health Services, WIC Program, 2019	As reported in citation	Cited reference	See reference	As reported	As reported in main text	As cited	See full reference citation	As cited
**83**	Davidson et al., 2024	USA	Implementation study	127 patients	Pediatric	Feeding disorders	Telehealth in pediatric feeding clinic	High satisfaction with telehealth services; caregivers found it more convenient than in-person visits; interest in continuing virtual visits	Moderate
**84**	Kodjebacheva et al., 2023	USA/International	Systematic review	Multiple studies	Pediatric	Various	Pediatric telehealth patient satisfaction	>80% satisfaction; convenience & time savings valued	High

**85**	Kaufman-Shriqui et al., 2021	Israel	Cross-sectional survey	98 dietitians	N/A (providers)	Various	Virtual nutrition consultation experiences	56% had technical difficulties; lessons from COVID	Moderate
**86**	Tong et al., 2022	USA	Retrospective cohort	154,538 encounters	Mixed ages	Various	Telehealth utilization & health disparities	Lower video use in low-income, minority populations	Moderate
**87**	Jones et al., 2022	USA	Repeated cross-sectional	1,045 parents	0-21 years	Various	Parent satisfaction with pediatric telehealth	Younger children (0-5) lowest satisfaction; COVID impact	Moderate
**88**	Kodjebacheva et al., 2023	International	Literature review	N/A	Children/adolescents	Various	Adolescent satisfaction with telehealth	Adolescents value privacy, flexibility; high satisfaction	Moderate
**89**	Rozga et al., 2021	USA	Cross-sectional survey	1,342 RDNs	N/A (providers)	Various	RDN telehealth use during COVID-19	Adoption increased 37% to 78%; training needs identified	Moderate

90	Bookari et al., 2023	10 Arab countries	Cross-sectional survey	1,476 dietitians	N/A (providers)	Various	Dietitian telenutrition practices & perspectives	Confidence increased 61% to 76.6%; importance recognized	Moderate
91	Gnagnarella et al., 2022	Italy	Cross-sectional survey	284 dietitians	N/A (providers)	Various	Telenutrition professional practice changes	Younger RDNs more likely to adopt; assessment concerns	Moderate
92	Alomar D, Almashmoum M, Eleftheriou I, Whelan P, Ainsworth J, 2024	As reported in citation	Review/systematic review	See reference	As reported	As reported in main text	As cited	See full reference citation	Moderate
93	Gustavson et al., 2023	USA	Implementation framework	N/A	N/A	Various	Strategies for equitable telehealth implementation	Leadership, resources, digital infrastructure critical	Moderate
94	Uscher-Pines et al., 2021	USA	Qualitative study	24 EDs	N/A (organizations)	Emergency telehealth	Telehealth innovations in emergency	Prior experience facilitated rapid	Moderate

							departments	expansion	
95	Mehta P, Stahl MG, Germone MM, Nagle S, Guigli R, Thomas J, Shull M, Liu E, 2020	As reported in citation	Cited reference	See reference	As reported	As reported in main text	As cited	See full reference citation	As cited
96	Shachar C, Engel J, Elwyn G, 2020	As reported in citation	Cited reference	See reference	As reported	As reported in main text	As cited	See full reference citation	As cited
97	Zhang Z, Bundorf MK, Gong Q, Shea CM, Gilleskie D, Sylvia SY, 2025	As reported in citation	Cited reference	See reference	As reported	As reported in main text	As cited	See full reference citation	As cited
98	Reference 98	As reported in citation	Cited reference	See reference	As reported	As reported in main text	As cited	See full reference citation	As cited
99	Saeed SA, Masters RM, 2021	As reported in citation	Cited reference	See reference	As reported	As reported in main text	As cited	See full reference citation	As cited

100	Price JC, Simpson DC, 2022	As reported in citation	Cited reference	See reference	As reported	As reported in main text	As cited	See full reference citation	As cited
101	HHS/ ASPE, 2021	USA	National survey	Population-level	Mixed ages	Various	Telehealth use disparities analysis	Video use: 68.8% (high-income) vs 38.1% (low-income)	High
102	Rural Health Info Hub	USA	Data report	N/A	N/A	Rural populations	Rural broadband access barriers	28% rural lack broadband vs 4% urban	Moderate
103	Joshi, 2023	USA	Commentary	N/A	N/A	Language barriers	Language access in telehealth	Professional interpretation in 200+ languages available	Low
104	Pullyblank et al., 2023	USA	Cross-sectional survey	1,128 patients	Mixed ages	Various	Telehealth access & preferences	Prior experience increases preference & comfort	Moderate
105	Kruse et al., 2017	International	Systematic review & meta-analysis	93 studies	Mixed ages	Various	Telehealth patient satisfaction	Majority positive experiences; high	High

								satisfaction	
106	Cueto V, Sanders LM, 2020	USA	Review	N/A (review)	Pediatric	Pediatric obesity - telehealth challenges	Telehealth for nutrition & exercise in COVID era	Inability to conduct in-person physical exams; reliance on caregiver-obtained measurements; clinical assessment limitations	Moderate
107	Chai et al., 2019	Australia	Validation study	163 parent-child dyads	2-5 years	General population	Parent-reported vs measured anthropometrics	BMI from parent-report has poor concordance	Moderate
108	Huybrechts et al., 2011	Belgium	Validation study	1,000 families	2-7 years	General population	Parent-reported vs measured height/weight	Parent measurement more accurate than estimation	Moderate
109	Einarsson et al., 2024	Norway	Qualitative study	20 dietitians	N/A (providers)	Various	Dietitian experiences with telenutrition assessment	Physical assessment limitations; visual aids help	Moderate

110	Kesari & Noel, 2023	USA	Clinical review	N/A	N/A	General	Nutritional assessment methods	Comprehensive physical exam needed for assessment	Moderate
111	Sheba Medical Center, 2020	Israel	Program description	50,000+ consultations	Mixed ages	Various	Sheba BEYOND virtual hospital	First Israeli virtual hospital; multidisciplinary	Low
112	Penn & Laron, 2023	Israel	Cross-sectional survey	519 Arab Israelis	Mixed ages	Various	Telehealth use & barriers in Arab population	Cultural & language barriers; connectivity issues	Moderate
113	Levi & Davidovitch, 2022	Israel	Health system report	N/A	N/A	Israeli healthcare system	Health care system overview	Universal coverage via 4 HMOs	Moderate
114	Grossman et al., 2020	Israel	Survey	141 pediatricians	N/A (providers)	Primary care pediatrics	Future of telemedicine post-COVID perceptions	Pediatricians see continued telehealth role	Moderate
